# Effect of increasing organic loading rate on hydrolytic enzyme activities and microbial communities during anaerobic digestion of energy crops

**DOI:** 10.3389/fmicb.2026.1894607

**Published:** 2026-08-12

**Authors:** Frederik Bade, Elliot Guerra-Blackmer, Alberto Meola, Simon Hellmann, Sören Weinrich, Lucie Moeller, Sabine Kleinsteuber

**Affiliations:** 1 Department Systemic Environmental Biotechnology, Helmholtz Centre for Environmental Research – UFZ, Leipzig, Germany; 2 Department of Microbial Biotechnology, Helmholtz Centre for Environmental Research – UFZ, Leipzig, Germany; 3 DBFZ Deutsches Biomasseforschungszentrum gemeinnützige GmbH, Leipzig, Germany; 4 Faculty of Mathematics and Computer Science, Leipzig University, Leipzig, Germany; 5 Professorship of Automatic Control and System Dynamics, Chemnitz University of Technology, Chemnitz, Germany; 6 Faculty of Energy, Building Services and Environmental Engineering, Münster University of Applied Sciences, Steinfurt, Germany

**Keywords:** 16S rRNA, amylase, chain elongation, esterase, *mcrA* genes, over-acidification, protease

## Abstract

**Introduction:**

Efficiency and stability of anaerobic digestion (AD) processes strongly depend on maintaining a balance between microbial conversion steps. Operational stress can disrupt this balance, leading to shifts in the microbial community, accumulation of intermediate metabolites, and changes in overall process performance. Therefore, the objective of the study was to better understand the role of hydrolysis and its interplay with subsequent conversion steps in the destabilization of the AD process during increasing organic loading rate (OLR).

**Methods:**

To systematically investigate the response of an AD process to operational stress, a stepwise increase in OLR until 15 g volatile solids (VS)/(L d) along with stepwise decrease of the hydraulic retention time (HRT) was applied in two parallel operated mesophilic lab-scale anaerobic digesters loaded with maize silage, cow manure and coarse-ground grain. The duplicate systems were comprehensively monitored, including key process parameters such as biogas production and composition, pH, buffer capacity, and volatile fatty acid (VFA) concentrations. Hydrolytic enzyme activities (amylase, protease, esterase) were measured to quantify key microbial functions, and microbial communities were analyzed via amplicon sequencing of 16S rRNA and *mcrA* genes to monitor compositional changes of the bacterial and methanogenic populations, respectively.

**Results and discussion:**

Both reactors showed similar trends in process performance until OLR was 2.5-fold increased, characterized by increasing hydrolytic enzyme activities and minor VFA accumulation. At higher OLR, VFA accumulation increased, and the process performance and microbial communities of the two reactors diverged. While methane production in one reactor collapsed due to over-acidification and was replaced by chain elongation as the predominant microbial process, the other reactor maintained methanogenic activity until 3-fold increase in OLR. These changes were also reflected in declining enzyme activities, where protease activity decreased first. Bacterial diversity in the reactor shifting to chain elongation decreased dramatically, indicating a loss of AD functionality under elevated OLR. These findings highlight the importance of microbial community composition and diversity for maintaining AD stability under increasing operational stress. Contrasting reactor responses suggest that even small differences in microbial communities or stochastic effects can influence process resilience.

## 1 Introduction

Renewable energies play an increasing role in Germany’s energy system, surpassing fossil-based electricity generation in 2025 for the third time in a row (Fraunhofer, 2026), with biogas representing an important component of this transition. However, the expansion of new anaerobic digestion (AD) plants, which increased significantly in the early 2000s, has slowed due to the changing economic conditions, including the expiration of subsidies under the German Renewable Energy Act, and the increased profitability of other renewable energy technologies (Daniel-Gromke et al., 2018). As a result, the focus of the biogas sector has shifted from base-load electricity supply to grid injection of biomethane, flexibilization of electricity production from biogas, substrate diversification, and increasing overall efficiency (Mertins and Wawer, 2022; Röder et al., 2022). To optimize the efficiency of AD, process stability is essential to maintain high biogas production and prevent process failure (Theuerl et al., 2019; Wu et al., 2021). This requires a thorough understanding of the key process parameters and their operational limits.

Increasing the organic loading rate (OLR) represents a controlled approach to challenge the stability limits of AD systems. When OLR is elevated, the balance between the different metabolic stages can be disrupted. This can lead to process instabilities, such as the accumulation of volatile fatty acids (VFAs), pH decline, loss in methane production, and, consequently, process failure (Schnürer and Jarvis, 2018; Wu et al., 2025).

AD consists of four stages—hydrolysis, acidogenesis, acetogenesis, and methanogenesis (Schnürer and Jarvis, 2018). Among these, hydrolysis is often the rate-limiting step, when complex, hardly degradable substrates such as agricultural residues are used. During hydrolysis, complex polymers such as polysaccharides, proteins, and lipids are broken down into simpler, soluble molecules, mainly through extracellular enzymes secreted by microorganisms, i.e., consortia of fermentative bacteria. In the subsequent acidogenesis, the formation of VFAs, alcohols, carbon dioxide (CO_2_), and hydrogen is performed by fermentative bacteria as well and cannot be separated from hydrolysis. Syntrophic bacteria are responsible for acetogenesis, which is the production of acetate from fermentation products, such as carboxylic acids and alcohols. The final step of AD produces methane and CO_2_ as the main components of biogas. The two main pathways are disproportionation of acetate into methane and CO_2_ (acetoclastic methanogenesis) and reduction of CO_2_ with hydrogen to methane (hydrogenotrophic methanogenesis). Both acetoclastic and hydrogenotrophic methanogenesis are performed exclusively by archaea. Methanogenic archaea consume the upstream products of AD but can also be inhibited by AD intermediates, making methanogenesis the rate-limiting step of AD. For instance, accumulation of ammonia resulting from nitrogen-rich feedstocks inhibits methanogens, and the AD process is impaired when acidogenesis products are not consumed fast enough by the methanogens, leading to over-acidification and thus further inhibition due to the accumulation of fatty acids (Schnürer and Jarvis, 2018).

Conventional AD monitoring relies on physicochemical parameters such as gas production, gas composition, pH, concentrations of VFAs and ammonium nitrogen, and buffer capacity [ratio of volatile organic acids (VOA) to total inorganic carbon (TIC)]. These indicators provide valuable information about process performance; however, they only reflect the chemical outcomes of microbial processes rather than the microbial activities itself. None of them is usable for monitoring the hydrolysis (Gasch et al., 2013). Hydrolysis can be assessed by activity measurement of extracellular hydrolytic enzymes. However, previous studies on monitoring hydrolytic enzyme activities in AD have mainly focused on two-stage AD (Gasch et al., 2013), often using alternative substrates such as sisal leaf (Mshandete et al., 2008), solid potato waste (Parawira et al., 2005), organic vegetable and flower waste (Zhang et al., 2007), and sludge from wastewater treatment plants (Odnell et al., 2016; Toja Ortega et al., 2021). To the best of our knowledge, only Xue et al. (2020) and Jensen et al. (2021) have investigated hydrolytic enzymes in single-stage AD treating lignocellulosic feedstocks, such as maize straw with liquid digestate recirculation, and wheat straw mixed with degassed reactor-specific slurry, respectively. Consequently, a knowledge gap exists regarding the role of hydrolytic enzymes in process stability in single-stage AD operated with energy-rich substrates, such as maize and grain in combination with manure, which are typical for full-scale AD plants in Central Europe.

AD is driven by complex and highly dynamic microbial communities, where functional stability depends on the balance between interacting functional groups (De Vrieze and Verstraete, 2016). Despite substantial variations in the microbial community composition between individual AD systems, methane production is maintained as a community function through functional redundancy (Langer et al., 2015; Lin et al., 2023). Nelson et al. (2011) investigated the global diversity of AD communities and identified a core microbiome from hundreds of AD communities around the world. High diversity of the microbial community contributes to a broader range of metabolic pathways, which may enhance resilience to environmental stress and support process stability (Carballa et al., 2015; De Vrieze and Verstraete, 2016; Li et al., 2024; Berninghaus and Radniecki, 2026). In contrast, low diversity and lack of functional redundancy may lead to disturbances in community function and directly impact the stability and productivity of the AD process, as an imbalance between functional groups can contribute to accumulation of intermediates such as VFAs and ammonia, ultimately causing process inhibition or failure (Shi et al., 2018).

The aim of this study was to investigate the role of hydrolysis in process destabilization during increasing OLR. Using two parallel reactors, operated with identical loading conditions, but exhibiting different process stability, we combined measurements of hydrolytic enzyme activities, microbial community composition, and conventional process parameters. While previous work has explored how AD microbial communities adapt to changing substrates, operational parameters, flexible feeding, and other factors known to impact AD stability and productivity (e.g., Krakat et al., 2011; Lv et al., 2014; Mulat et al., 2016, Weithmann et al., 2021), only few studies have investigated community adaptation and change in response to organic overload. This study therefore aimed to link hydrolytic functions and community dynamics to process performance for a better understanding of resilience to overloading and acidification.

## 2 Materials and methods

### 2.1 Reactor operation and sampling

The experiments were carried out in two 15-L continuous stirred tank reactors (CSTRs) with an initial working volume of 12 L. A schematic diagram of the CSTRs is shown in Supplementary Figure 1. The CSTRs were operated in parallel, i.e., as biological replicates, under mesophilic conditions (40 ± 1 °C) and continuously stirred with an initial speed of 90 rpm. The inoculum was obtained from a mesophilic full-scale research biogas plant, a CSTR digesting maize silage. During the experimental period, the reactors were loaded identically once a day with coarse-ground grain, maize silage and cattle manure with a step-wise increasing OLR from 4.5 g volatile solids (VS)/(L d) to 15 g VS/(L d). A detailed overview of the OLR, used substrates, and hydraulic retention time (HRT) is given in Table 1. Prior to the described experimental period, the reactors had been operated under identical conditions for 847 days, but with additional substrates (grass silage, straw, and distillers’ dried grains with solubles) in addition to the substrates used in the present study. Furthermore, the OLR had varied between 1.5 and 5 g VS/(L d) during this prior period, and the HRT had been kept mostly at 40 days.

**TABLE 1:** Overview on substrate loading plan, including organic loading rates (OLR) of individual substrates and hydrolytic retention time (HRT) during the experimental period.

Time [d]	OLR				HRT [d]
	OLR [g VS/(L d)]	Cattle manure [g VS/(L d)]	Maize silage [g VS/(L d)]	Grain [g VS/(L d)]	
0–7	4.5	1.3	1.2	2	35.7
8–13	5.5	1.7	2.8	1	26.7
14–20	6.5	2.1	3.4	1	24.3
21–27	7.5	2.1	4.3	1.1	24.1
28–34	8.5	2.3	5.1	1.1	21.7
35–41	9.5	2.9	5.4	1.2	18.2
42–47	10.5	3.3	5.9	1.3	16.6
48–55	11.5	3.6	7.9	0	14.6
56–69	12.5	3.9	8.6	0	13.5
70–77	12.5	3.6	8.9	0	13.4
78–97	14	3.9	10.1	0	12.4
98–115	15	4.3	10.7	0	11.6
116–126	0	0	0	0	0

The actual OLR slightly deviated as indicated in the respective figures.

A BlueVCount device (BlueSens, Germany) was used to measure the cumulative volume of the produced biogas. Biogas quantities were normalized to standard temperature and pressure. Produced biogas was continuously analyzed for CH_4_ and CO_2_ by use of a BlueVary online gas analyzer (BlueSens, Germany), and additionally collected in a gas bag downstream of the BlueSens devices for offline composition analyses. To this end, gas composition (CH_4_, CO_2_, H_2_S, H_2_, N_2_, O_2_) was measured by use of an Awite Awiflex gas analyzer (Awite Bioenergie, Germany). Reactor temperature was measured with a thermometer (Almemo 2390-1, Germany).

Due to extensive foaming, the working volume was reduced to 9 L on day 70 of the experiment, and 6–10 mL of an antifoaming agent (ANTIFOAM BTX2, Van Meeuwen FoodCare^®^, Netherlands) were added per day. To mitigate foaming and increased viscosity at higher OLR, the stirring speed was increased over the experimental time, to 110 rpm after day 28, to 130 rpm after day 49, and to 150 rpm after day 52, where it remained until the end of the experiment.

In addition to online monitoring, digestate samples were collected before substrate loading and centrifuged for analysis of dissolved metabolites. VOA/TIC and pH were determined five times and ammonium nitrogen once per week. Samples for fermentation products (VFAs and ethanol) were taken five times per week, prepared for analysis as described in section 2.2 (centrifugation, followed by the addition of an internal standard, methanol, and sulfuric acid to the supernatant), and stored at circa 7 °C until analysis. Samples for enzyme assays and microbiome analysis were taken once or twice per week. Digestate samples for microbiome analyses were immediately frozen at –40 °C until DNA extraction was performed.

### 2.2 Analytical methods

Concentrations of VFAs (C1-C7) were analyzed according to Schumacher et al. (2019) by use of a 7890A gas chromatograph with a flame ionization detector (FID; Agilent, United States). Samples for VFA analysis were taken from the liquid phase of the digestate after solids had been removed by centrifugation at 10,000 × g for 10 min. For analysis, 3 mL of the sample, 1 mL of the internal standard (2-methylbutyric acid at 184 mg/L), 0.5 mL of methanol, and 2.5 mL of diluted sulfuric acid (1:5) were placed in a 20-mL headspace vial. The vial was then tightly closed with an aluminum crimp cap containing a PTFE/silicone septum using a crimper. The samples were stored at 4 °C until the measurement. Headspace sampling was performed using a Turbomatrix 110 autosampler (PerkinElmer, United States). The injection mode of the gas chromatograph was split 1:10. The injector temperature was maintained at 220 °C, and a DB-FFAP column (60 m, 0.25 mm x 0.5 mm; Agilent, United States) was used for the separation. Gas chromatography was operated using nitrogen as the carrier gas and in a programmed temperature mode as follows: Beginning with a temperature of 40 °C for 20 min, followed by a linear ramp of 10 K/min until reaching 200 °C, which was held for 10 min. A deactivated transfer line with an inner diameter of 0.25 mm and a FID (Agilent, United States) heated at 260 °C were used for data acquisition. The headspace sampler settings were as follows: temperature: oven 85 °C, needle 100 °C, transfer line 110 °C; timing: vial equilibration: 25 min; pressurization: 3.0 min; injection: 0.4 min; dwell time: 0.5 min. The pressure settings were as follows: carrier: 32.0 psi; vial: 28.5 psi.

Ammonium nitrogen, pH, and the ratio of VOA/TIC were analyzed using analytical procedures as previously described by Strach (2020) and Strach and Zechendorf (2020), respectively.

### 2.3 Hydrolytic enzyme assays

Activities of selected hydrolytic enzymes—amylase, protease, and esterase—were measured according to Gasch et al. (2013).

Digestate samples were centrifuged twice at 10,000 × g for 10 min at 4 °C. Supernatant was collected and stored on ice until the start of the enzyme assays. All enzyme tests were performed in triplicate and sample blanks were taken for each sample.

Amylase activity was determined by measuring reduced sugars with dinitrosalicylic acid reagent according to Gasch et al. (2013). A 1% solution of soluble starch was dissolved in 0.2 M potassium phosphate buffer (pH 6.5). A volume of 350 μL of the prewarmed starch solution was mixed at 750 rpm with 150 μL of supernatant and incubated at 50 °C in a thermomixer (Thermomixer Compact, Eppendorf, Germany). After 1 h of incubation, the reaction was stopped by adding 750 μL dinitrosalicylic acid reagent, consisting of 1% (w/v) dinitrosalicylic acid, 0.2% (v/v) phenol, 0.05% (w/v) sodium sulfite, 20% (w/v) potassium sodium tartrate, and 1% (w/v) sodium hydroxide. The reaction mixture was then heated for 15 min at 100 °C. Subsequently, the test tubes were cooled on ice and centrifuged at 10,000 × for 5 min. Absorbance of the supernatant was measured at 575 nm using an UV-1601 spectrophotometer (Shimadzu, Japan) compared to a blank value. Amylase activity was calculated using a standard curve prepared with D-glucose.

Protease activity was measured using the azocasein method (Gasch et al., 2013). A 0.5%-azocasein solution was prepared in 0.2 M Tris-HCl buffer (pH 7.5). An equal volume of 0.5 mL prewarmed azocasein solution and 0.5 mL supernatant were mixed and incubated for 1 h at 50 °C. The reaction was stopped by addition of 1 mL 10% (w/v) trichloroacetic acid. The samples were centrifuged at 10,000 × g for 5 min, and absorbance was measured at 380 nm (UV-1601 spectrophotometer, Shimadzu, Japan) against a blank. Protease activity was calculated using a standard curve prepared with the enzyme papain, whereby 1 mg/L papain corresponds to 30 international units (IU).

Esterase activity was measured using 2% fluorescein diacetate in acetone as a substrate (Gasch et al., 2013). A total of 9.4 mL 0.2 M Tris-HCl buffer (pH 7.5) was mixed with 0.1 mL fluorescein diacetate solution and with 0.5 mL supernatant. The mixture was incubated at 30 °C for 1 h. Absorbance was measured at 490 nm (UV-1601 spectrophotometer, Shimadzu, Japan) against a blank. Overall hydrolytic activity was calculated using a standard curve prepared with pure fluorescein as a product of the hydrolytic reaction.

### 2.4 Microbial community analysis

DNA was extracted from 400 to 500 mg of frozen digestate samples with a NucleoSpin Soil DNA extraction kit (MACHEREY-NAGEL GmbH & Co., Germany) following the manufacturer’s instructions. The extracted DNA was quantified with Qubit dsDNA BR Assay Kit (Thermo Fisher Scientific, United States) and stored at –20 °C for later use. Aliquots for sequencing were diluted to 5 ng/μL.

Bacterial diversity was analyzed by amplicon sequencing of 16S rRNA genes (V3/V4 regions) using the primer set 341f (5’-CCT ACG GGN GGC WGC AG-3’) and 785r (5’-GAC TAC HVG GGT ATC TAA KCC-3’) as described by Klindworth et al. (2013). Methanogenic archaea diversity was analyzed by amplicon sequencing of the *mcrA* genes (endoding the methyl coenzyme M reductase alpha subunit) using the primers mlas (5’-GGT GGT GTM GGD TTC ACM CAR TA-3’) and *mcrA*-rev (5’-CGT TCA TBG CGT AGT TVG GRT AGT-3’) as described by Steinberg and Regan (2008). Amplicon libraries were prepared following the Illumina manual for 16S Metagenomic Sequencing Library Preparation and sequenced on the Illumina MiSeq platform with the MiSeq reagent kit v3 with 2 × 300 cycles (Illumina, United States).

The demultiplexed raw reads were deposited at the EMBL European Nucleotide Archive (ENA) under the study accession number (PRJEB113461).

Sequence data analysis was performed in R (version 4.3.1). The raw reads produced by Miseq sequencing of the amplicon libraries were processed with the Dada2 (version 1.28.0) R package (Callahan et al., 2016). During this processing chimeras were removed and paired ends were merged. For 16S rRNA, the forward and reverse reads were truncated to 270 base pairs (bp) and 240 bp, respectively. The *mcrA* reads were truncated to 270 and 230 bp, respectively. This truncation was made according to the respective read quality profiles. Primer sequences were removed with cutadapt (version 5.0; Martin, 2011). For taxonomy assignment, the RDP naive Bayesian classifier contained in the Dada2 package was trained on the silva_nr99_v138.2 training dataset, and taxonomy of the 16S rRNA amplicon sequencing variants (ASVs) was assigned using the Silva database (version 138.2; Quast et al., 2012). Archaeal reads were removed from the dataset since the primers do not cover all archaea (Klindworth et al., 2013). Taxonomy of the *mcrA* ASVs was assigned using the RDP naive Bayesian classifier as well, but trained on the *mcrA* database described by Logroño et al. (2020), which is based on the FunGene database (Fish et al., 2013).

For further processing of the ASV datasets and visualization of microbial community composition and diversity, the R package Phyloseq (McMurdie and Holmes, 2013) was used. This processing removed low abundance ASVs that appeared less than five times per sample or in less than seven samples. After removing the archaeal reads from the 16S rRNA dataset, read counts were normalized to the lowest read count in each library, being 13,050 reads for 16S rRNA and 2,444 reads for *mcrA*.

Additional R packages used for ASV data processing and visualization included ShortRead (Morgan et al., 2009), Biostrings (Pagès et al., 2024), vegan (Oksanen et al., 2025), janitor (Firke, 2024), and RColorBrewer (Neuwirth, 2022).

### 2.5 Statistical analyses

Spearman correlation analyses were performed in R (version 4.3.1) with the tidyverse collection of packages (Wickham et al., 2019). Correlation analyses between the VOA/TIC ratio and enzyme activities were restricted to measurements from day 34 onward and to VOA/TIC ratios below 1. *P*-values were adjusted for multiple testing using the Benjamini-Hochberg false discovery rate (FDR) procedure.

Spearman correlations between the ASV data and abiotic parameters including enzyme activities were analyzed after relative ASV abundances had been normalized with a centered log ratio (CLR) transformation using the R package SpiecEasi (Kurtz et al., 2026). The threshold for significance in these correlations was defined as a *p*-value lower than 0.01 and an absolute Spearman’s rho (ρ) value of greater than 0.3.

Permutational multivariate analysis of variance (PERMANOVA) was conducted using the adonis2 function in the R package vegan with 999 permutations to test the significance of experiment duration on microbial community composition. Significance was assessed using permutation-based *p*-values lower than 0.01, and effect sizes were reported as R^2^ values.

## 3 Results

### 3.1 Effect of increasing OLR on physicochemical process parameters

**FIGURE 1:**
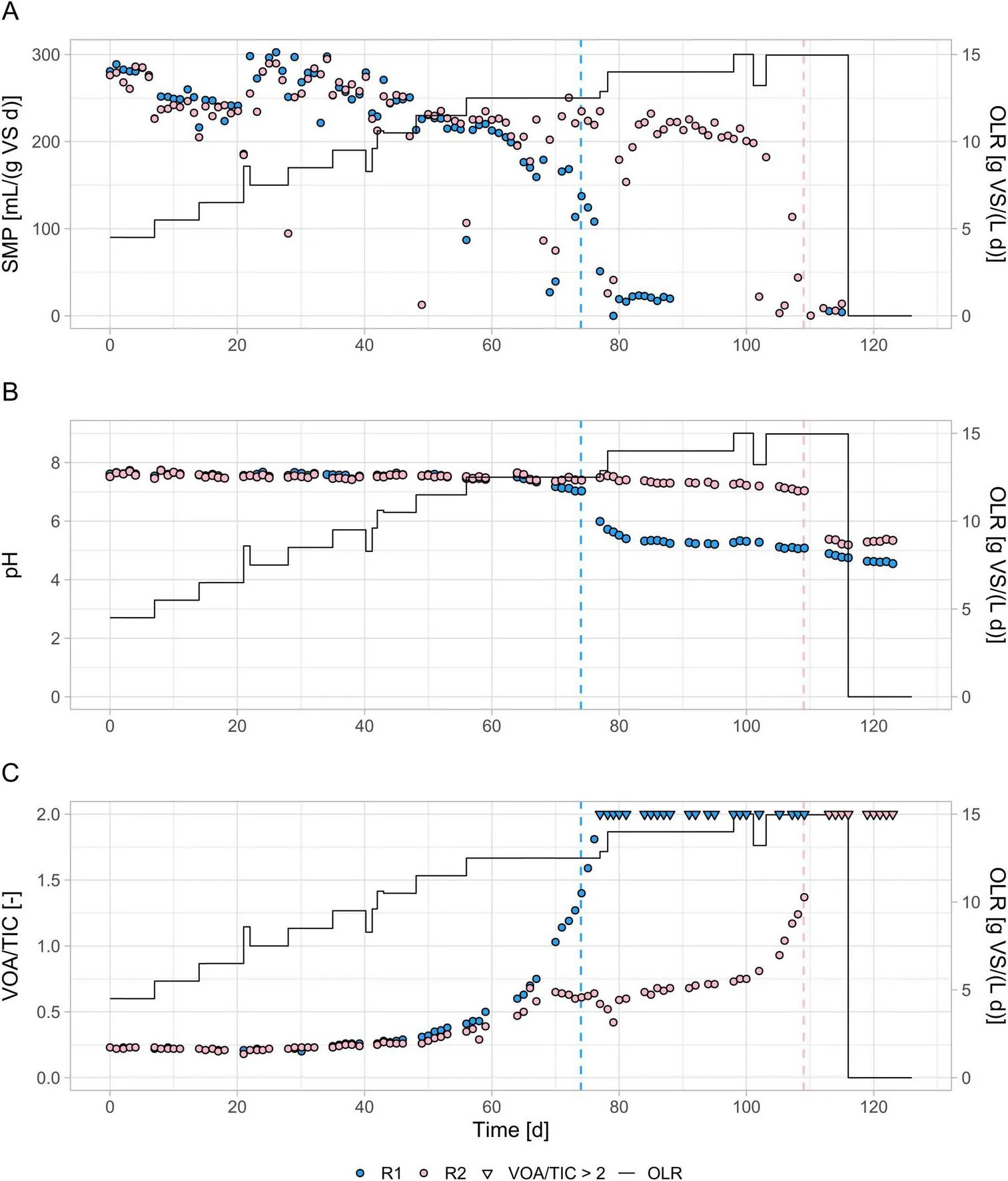
Time courses of specific methane production (SMP; **A**), pH **(B)**, and the volatile organic acid to total inorganic carbon (VOA/TIC) ratio **(C)** in the two parallel continuous stirred tank reactors (R1 and R2) during gradual increase of the organic loading rate (OLR). Dashed vertical lines represent the onset of over-acidification, defined as the last sampling point with pH > 6.0 (blue line: R1, pink line: R2).

The time courses of specific methane production, pH value, and VOA/TIC ratio in the two parallel AD reactors are presented in Figure 1. The ammonium nitrogen concentration showed no considerable changes throughout the experiment (Supplementary Figure 2).

Up to an OLR of 11.5 g VS/(L d), process parameters in both reactors responded similarly to gradual OLR increase and stayed in their optimum ranges. The specific methane production was comparable in both reactors, ranging from approximately 210 to 300 mL/(g VS d) (Figure 1A), the pH was constant at approximately 7.6 (Figure 1B), and the VOA/TIC ratio was below 0.3 (Figure 1C). Both reactors showed two maxima in specific methane production. The first maximum of 280 mL/(g VS d) occurred at an OLR of 4.5 g VS/(L d) at the beginning of the experimental period. The second maximum was observed at an OLR between 7.5 and 9.5 g VS/(L d) in the period between days 22 and 34, with values slightly higher than 280 mL/(g VS d). Overall maximum values were 302 mL/(g VS d) on day 26 in R1, and 295 mL/(g VS d) on day 34 in R2. Outliers in the specific methane production were caused by extensive foaming events, which led to clogging of the gas pipes.

Further increase in OLR to 12.5 g VS/(L d) caused different effects in the two reactors. In R1, sharp declines in specific methane production and pH were observed. Methane production almost stopped, and the pH dropped below 6.0 after day 74 and further to a final value of 4.6 on day 126. The increasing OLR also caused an increase in VOA/TIC ratio to values above 0.6 after day 59, and further until a maximum of 25.6 on day 107. In contrast to R1, specific methane production remained unaffected in R2, although the VOA/TIC ratio also exceeded 0.6 on day 66. At an OLR of 15 g VS/(L d), R2 also exhibited a drop of specific methane production and pH. The pH dropped below 6.0 after day 106 and further down to a final value of 5.3 on day 126, ultimately reflecting a process failure. A maximum VOA/TIC ratio of 10.5 was reached in R2 on day 116. Although both reactors were operated in parallel, methane production in R1 collapsed already at an OLR of 12.5 g VS/(L d), whereas specific methane production in R2 first remained unaffected, slightly decreased after day 80 and sharply declined on day 100 at an OLR of 15 g VS/(L d), corresponding to an increase of VOA/TIC to > 0.6.

**FIGURE 2:**
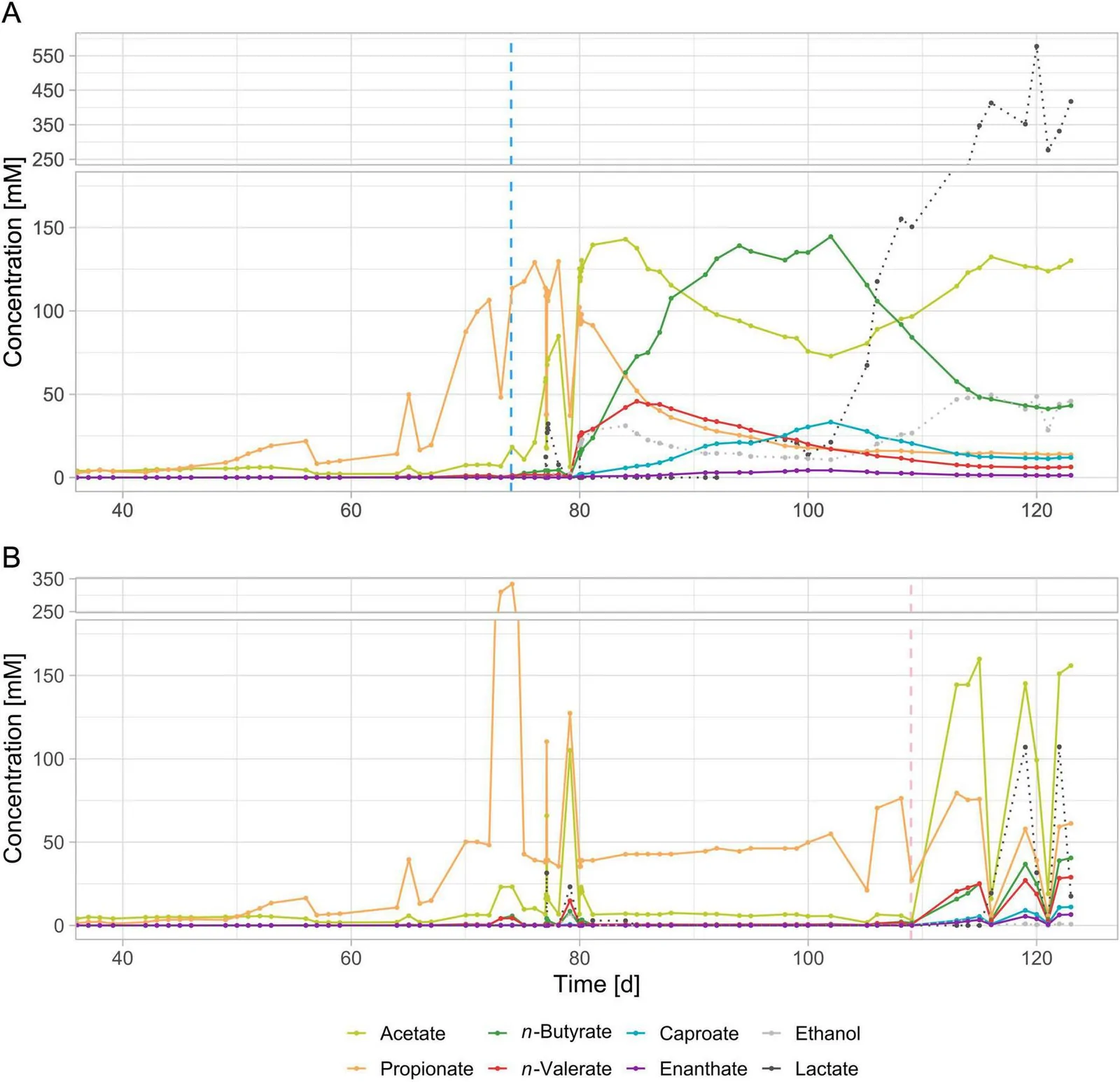
Concentrations of carboxylic acids and ethanol in the two parallel continuous stirred tank reactors R1 **(A)** and R2 **(B)** after day 40. Dashed vertical lines represent the onset of over-acidification, defined as the last sampling point with pH > 6.0 (blue line: R1, pink line: R2).

Individual fermentation products are considered more specific indicators for process instabilities in AD, as their concentrations provide more detailed information on the metabolic processes leading to over-acidification than the VOA/TIC ratio. Figure 2 shows the time courses of the concentrations of the main AD intermediates acetate, propionate, *n*-butyrate, *n*-valerate, caproate, enanthate, lactate, and ethanol after day 40.

In accordance with the earlier acidification and process failure, higher VFA concentrations were observed in R1 (Figure 2A) compared to R2 (Figure 2B), and propionate was the first of all VFAs to accumulate. Until day 70, the propionate concentrations showed a similar trend in both reactors. Thereafter, propionate accumulated in R1 up to a maximum of 129.7 mM on day 78. After its maximum, the concentration decreased to a final concentration of 13.7 mM. In R2, greater fluctuations in propionate concentration were observed, with a maximum of 334.4 mM on day 74. However, compared to R1, the propionate concentration remained higher with approximately 41.0 mM for a longer period before it increased to a second peak of 79.4 mM at an OLR of 15 g VS/(L d) on day 113.

Once the propionate concentration in each reactor reached its maximum, acetate began to accumulate. In R1, the acetate concentration reached its peak of 143.0 mM on day 84, whereas in R2, the maximum of 159.9 mM was reached on day 115. Subsequently, the acetate concentration decreased to 72.8 mM in R1 on day 102 and increased again to a second peak of 133.4 mM toward the end of the experimental period on day 116. No second peak was observed in R2, with acetate remaining at approximately 150.0 mM until the end of the experiment.

Accumulation of *n*-butyrate, *n*-valerate, and ethanol began in parallel around day 80 in R1 and day 113 in R2, when pH drop below 6 had already occurred in each reactor. In R1, the maximum *n*-butyrate concentration of 144.6 mM was observed on day 102, coinciding with a local minimum of acetate. Thereafter, *n*-butyrate concentration decreased to a final value of 43.2 mM. In R2, *n*-butyrate accumulated toward the end of the experiment, reaching its maximum concentration of 38.9 mM on day 123. The highest *n*-valerate concentration of 45.8 mM occurred in R1 on day 85. Ethanol exhibited two peak concentrations: the first at a concentration of 31.1 mM g/L on day 84, and the second toward the end of the experimental period when it remained at approximately 47 mM from day 114 onwards. In R2, the maximum concentration of *n*-valerate was 28.9 g/L on day 123, while the highest ethanol concentrations were only 7.1 mM on day 79 and 5.1 mM on day 115.

Caproate and enanthate accumulated also in parallel. In R1, caproate peaked on day 102 at a concentration of 33.3 mM. In R2, caproate reached 11.1 mM on day 123. Enanthate occurred in comparably low concentrations of 4.3 mM on day 100 in R1, and 6.54 mM on day 123 in R2.

The fermentation product that showed the highest recorded concentrations was lactate: In R1, two smaller peaks of 32.2 mM on day 77 and 13.6 mM on day 80 occurred. Ongoing feeding during over-acidification resulted in an exponential increase in lactate concentrations until reaching a maximum of 577.1 mM on day 120. In R2, lactate concentrations also showed two peaks of 31.5 mM on day 77 and 23.2 mM on day 79 and increased toward the end of the experiment, reaching a maximum concentration of 107.2 mM on day 123.

Furthermore, elevated concentrations of the phenyl carboxylic acids phenylacetate and phenylpropionate were observed (Supplementary Figure 3). Phenylacetate began to accumulate around day 30 and showed a maximum in both reactors on day 74 of 5.1 mM in R1 and 9.7 mM in R2. Phenylpropionate concentration also peaked in both reactors on day 74 (5.7 mM in R1 and 6.7 mM in R2).

### 3.2 Effect of increasing OLR on hydrolytic enzyme activities

Based on the substrate composition (maize silage, grain, cow manure) used for this experiment, amylase and protease were chosen to assess the hydrolysis phase. Amylases catalyze the degradation of starch, the primary carbohydrate in maize and grain, whereas proteases catalyze the degradation of proteins, a major fraction of the organic nitrogen in the substrates. Additionally, the hydrolysis of fluorescein diacetate by extracellular enzymes was determined. This assay is often referred to as an esterase assay (Obst, 1985; Gasch et al., 2013). However, fluorescein diacetate is hydrolyzed by various enzymes, including proteases, lipases, and esterases (Schnürer and Rosswall, 1982). Therefore, Obst (1985) proposed using this assay as an indicator of total heterotrophic activity. However, the term “esterase activity” is used for simplicity.

**FIGURE 3:**
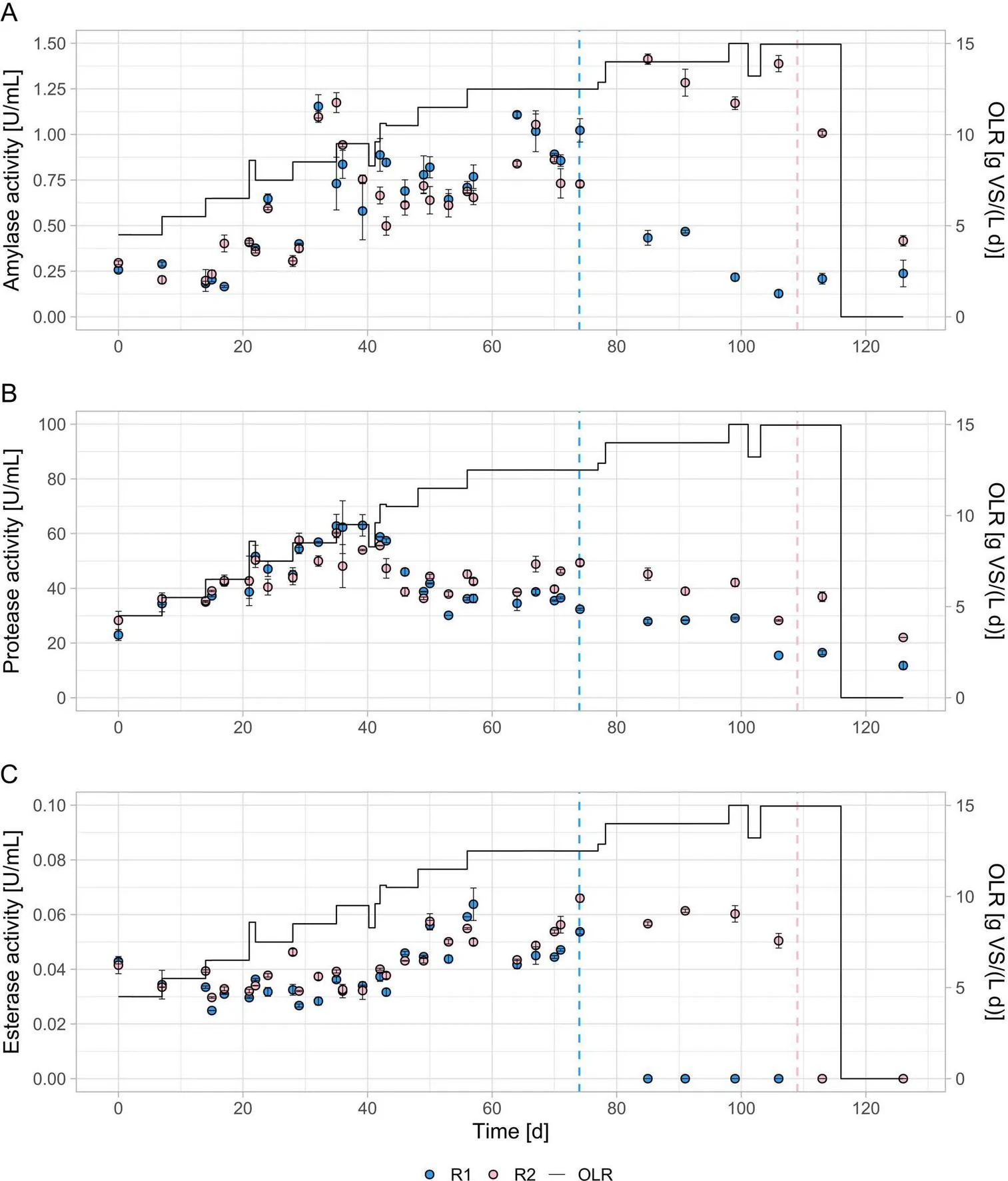
Time courses of the hydrolytic enzyme activities of amylase **(A)**, protease **(B)**, and esterase **(C)** in the two continuous stirred tank reactors (R1 and R2) along the gradual increase in organic loading rate (OLR; *n* = 3). Dashed vertical lines represent the onset of over-acidification, defined as the last sampling point with pH > 6.0 (blue line: R1, pink line: R2).

Figure 3 illustrates the activities of amylase (Figure 3A), protease (Figure 3B), and esterase (Figure 3C) over the experimental period. In general, as the OLR was gradually increased, the activities of all three enzymes also rose.

Amylase activity started at 0.26 ± 0.01 U/mL in R1 and at 0.30 ± 0.01 U/mL in R2. It increased in both reactors until reaching a first maximum on day 31 in R1 of 1.15 ± 0.05 U/mL and on days 32 and 35 in R2 of 1.10 ± 0.02 U/mL and 1.17 ± 0.04 U/mL, respectively, coinciding with periods of high specific methane production. Subsequently, a transient decline was observed, followed by a renewed increase until 1.11 ± 0.01 U/mL on day 64 and at an OLR of 12.5 g VS/(L d) in R1 and 1.41 ± 0.02 U/mL on day 85 at an OLR of 14 g VS/(L d), and 1.39 ± 0.04 U/mL on day 106 at an OLR of 15 g VS/(L d) in R2. During over-acidification, amylase activity decreased sharply. In R1, a decrease in amylase activity was observed after day 74, reaching a final activity of 0.23 ± 0.06 U/mL. In R2, a drop in amylase activity occurred after day 109 to a final activity of 0.42 ± 0.02 U/mL.

Protease activity also increased proportionately with rising OLR in both reactors. In R1, it started at 22.96 ± 1.65 U/mL and almost tripled to its maximum of 63.03 ± 3.21 U/mL on day 39 and at an OLR of 9.5 g VS/(L d). Protease activity in R2 started at values of 28.26 ± 2.72 U/mL, and although over-acidification occurred later, the highest activity of 60.25 ± 0.19 U/mL was detected on day 35 at an OLR of 9.5 g VS/(L d), similar to R1. The maximum protease activities in both reactors coincided with high specific methane production and declined continuously from this point onward at OLR higher than 9.5 g VS/(L d). The decline was steeper in R1 than in R2, leading to final protease activities of 11.76 ± 1.17 U/mL and 22.03 ± 0.00 U/mL, respectively (Figure 3B).

In both AD reactors, esterase activities rose with the increasing OLR up to 12.5 g VS/(L d). In R1, they increased from an initial activity of 0.04 ± 0.00 U/mL to a maximum of 0.06 ± 0.00 U/mL on day 57. In R2, they rose from 0.04 ± 0.00 U/mL to 0.07 ± 0.00 U/mL on day 71. In contrast to amylase and protease activities, no esterase activity was detected after the pH drop below 6, i.e., after day 85 for R1 and day 113 for R2 (Figure 3C).

To evaluate the relationship between extracellular hydrolytic enzyme activities and process stability, Spearman correlation analysis was performed between the VOA/TIC ratio and activities of amylase, protease, and esterase. Correlations were limited to data points obtained after day 34, the time point of the highest specific methane production, and to VOA/TIC values below 1, to avoid bias from extreme process failure conditions. The Spearman correlation analysis (amylase activity: ρ = 0.25, *p* = 0.42 for R1 and ρ = 0.36, *p* = 0.26 for R2; protease activity: ρ = –0.88, *p* < 0.01 for R1 and ρ = –0.35, *p* = 0.15 for R2; esterase activity: ρ = 0.68, *p* = 0.011 for R1 and ρ = 0.83, *p* < 0.01 for R2) showed significant correlations for the protease activity in R1, and for the esterase activity in both reactors. Although not significant in R2, only protease activities showed negative correlations with VOA/TIC, i.e., protease activity decreased with increasing VOA/TIC values (Supplementary Figure 4).

### 3.3 Effect of increasing OLR on microbial communities

**FIGURE 4:**
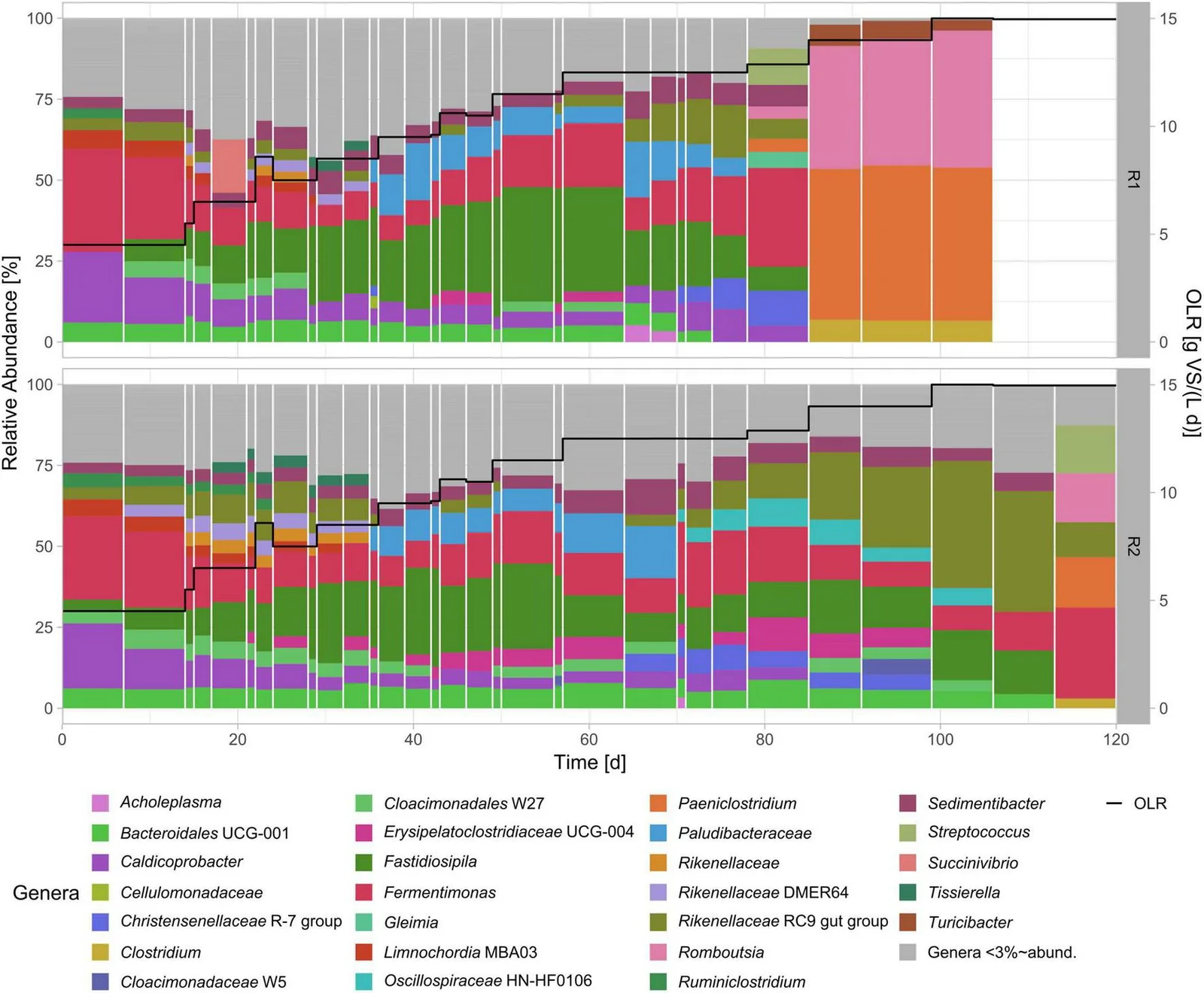
Bacterial community composition based on 16S rRNA amplicon sequence variants (ASVs). ASVs are agglomerated at the genus level. The complete ASV table showing the sequence, relative abundance, and taxonomy of each ASV can be found in Supplementary Table 1. The last two data points for R1 are missing due to insufficient DNA amounts extracted from these samples. The organic loading rate (OLR) appears different from previous figures due to the visualization of only the OLR values corresponding to the sampling times.

**FIGURE 5:**
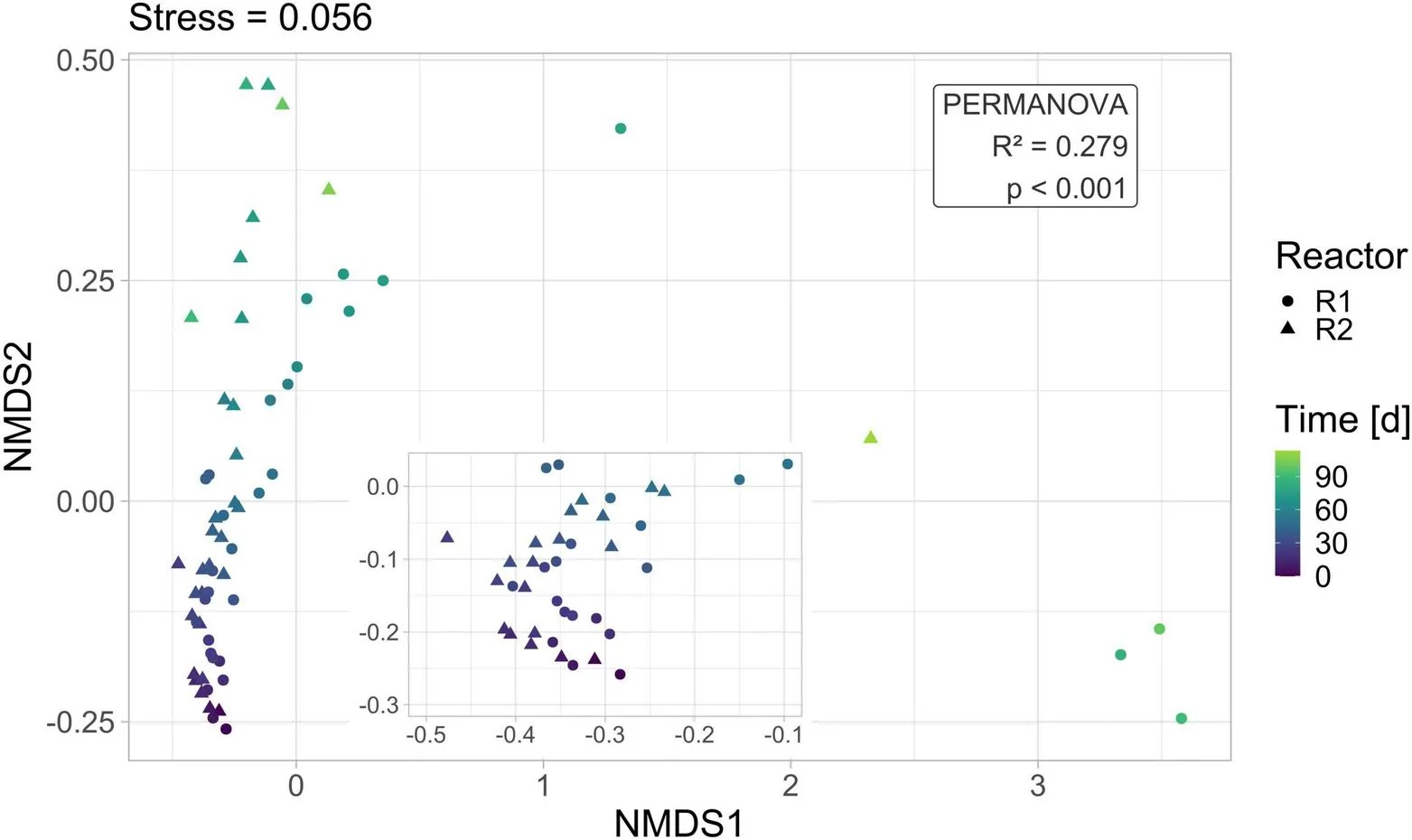
Non-metric multidimensional scaling (NMDS) plot of Bray-Curtis dissimilarity of bacterial communities based on 16S rRNA amplicon sequence variants (ASVs). The insert shows the data points representing samples taken until day 50.

At low OLR, bacterial communities in both reactors were dominated by the genera *Fermentimonas* and *Caldicoprobacter* (Figure 4). With increasing OLR, *Fastidiosipila* was initially enriched in both reactors, reaching its highest relative abundance of 37% on day 49 in R1 and 30% on day 42 in R2. Its relative abundance then decreased while remaining extant until falling below the 3% threshold after methanogenesis in each reactor collapsed, i.e., on days 85 and 113 for R1 and R2, respectively. Late sampling points from both reactors show dramatic changes in bacterial community composition alongside increasing OLR, with an abrupt community shift after day 78 in R1. Reactor R2 showed a delay in community shift of 26 days compared to R1, corresponding with the AD process remaining intact in that reactor for a longer time. In addition, the bacterial community in R2 shifted to ASVs associated with the *Rikenellaceae* RC9 gut group at high OLR (up to 15 g VS/[L d]). This genus reached its highest relative abundance of 39% in R2 on day 106. In R1, ASVs associated with the *Rikenellaceae* RC9 gut group also gained in relative abundance with the highest proportion of 16.2% on day 74. At this time point, critical acidification started in R1 with the pH dropping below 6. The major bacterial community shift in R1 took place directly after this onset of acidification and corresponded to AD process failure and a significant reduction of methane production. In both reactors, *Romboutsia* and *Paeniclostridium* substantially gained in relative abundance during this over-acidification period. The diverging trends in community dynamics and the abrupt community shifts upon over-acidification are also reflected in the beta diversity of the bacterial communities (Figure 5). The NMDS plot shows parallel community development until AD process failure in R1, as illustrated by data points from both reactors trending together with time as the experiment continued. In accordance with the bar plots (Figure 4), the two communities diverged substantially for the last four data points from R1. However, the divergent development of both communities started already earlier around day 74, as can be seen in Figure 5.

Alpha diversity metrics based on 16S rRNA ASVs shows a general downward trend in bacterial diversity in both reactors (Supplementary Figure 5). Richness (observed ASVs) as well as Shannon and Simpson indices all decrease over time, with reactor R1 showing a steeper decline than R2. The bacterial community in R2 maintained a higher diversity throughout the experimental time. Diversity remained higher until the very last data point, whereas the last three data points for R1 indicate an abrupt decline of richness and evenness, in accordance with the trends seen in the bar plots (Figure 4) and the NMDS plot (Figure 5).

**FIGURE 6:**
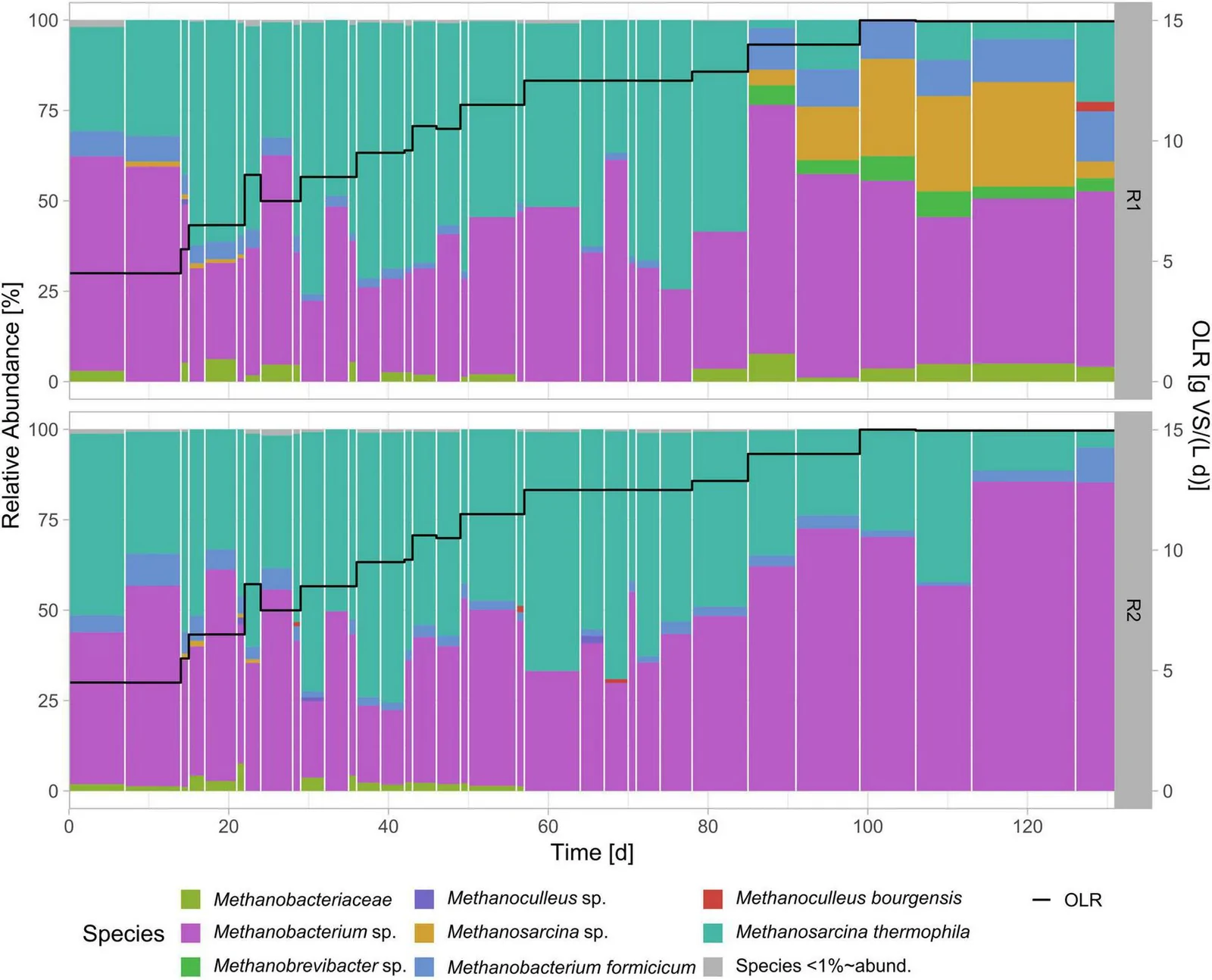
Community composition of methanogenic archaea based on *mcrA* amplicon sequence variants (ASVs). ASVs are agglomerated at the species level where possible, or to the next taxonomic level where no species classification was possible. The complete ASV table showing the sequence, relative abundance, and taxonomy of each ASV can be found in Supplementary Table 1. The organic loading rate (OLR) appears different from previous figures due to the visualization of only the OLR values corresponding to the sampling times.

The community of methanogenic archaea was substantially less diverse than their bacteria counterparts. As seen by the *mcrA* ASV data plotted in Figure 6, both reactors’ methanogenic communities were dominated by ASVs associated with *Methanosarcina thermophila* and *Methanobacterium* sp. *M. thermophila* showed a high relative abundance, with a maximum of 75% on day 29 and 74% on day 39 in R1 and R2, respectively. The methanogenic community in R1 dramatically shifted between days 78 and 85, characterized by a decrease in *M. thermophila* relative abundance from 58 to 2.1%. A similar decrease of *M. thermophila* relative abundance occurred in R2, but later and not as pronounced. By day 126, *M. thermophila* only represented 4.3% of total ASVs in R2. In contrast to *M. thermophila*, *Methanobacterium* sp. stayed dominant in both reactors throughout the experiment. In addition, a substantial increase in relative abundance of *Methanobacterium formicicum* and other ASVs associated with the genera *Methanosarcina* and *Methanobrevibacter* was observed in R1 after the OLR increase to 14 g VS/(L d). ASVs associated with other *Methanosarcina* sp. reached a peak of 29% on day 113 in R1, while *M. formicicum* reached its highest share with 14% on day 126. A small population of *Methanoculleus bourgensis* was detected in both reactors as well, reaching a relative abundance of 2.6% on day 126 and 1.7% on day 56 in R1 and R2, respectively.

Beta diversity of the methanogenic communities (Supplementary Figure 6) followed a similar trend as the bacterial communities. Initial data points clustered together until day 85, after which the methanogenic communities diverged. This corroborates the general trend visible in the *mcrA* ASV bar plot (Figure 6).

### 3.4 Correlation of process parameters with microbial community dynamics

**FIGURE 7:**
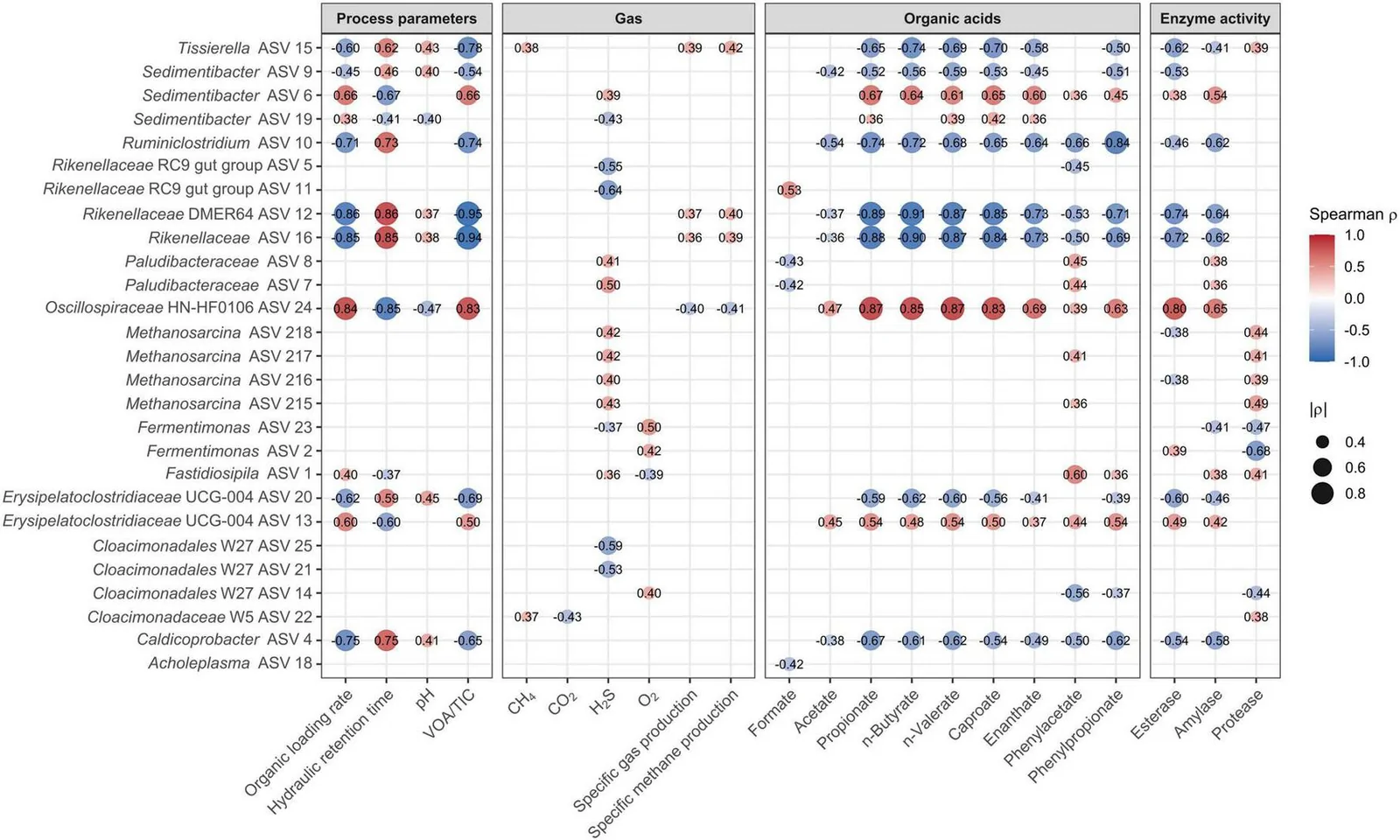
Spearman correlations between the most abundant amplicon sequence variants (ASV) and abiotic parameters at volatile organic acid to total inorganic carbon (VOA/TIC) ratio < 1.0. Significance was defined at *p* < 0.01. Only correlations with a Spearman correlation coefficient ρ > | 0.3| are shown.

**FIGURE 8:**
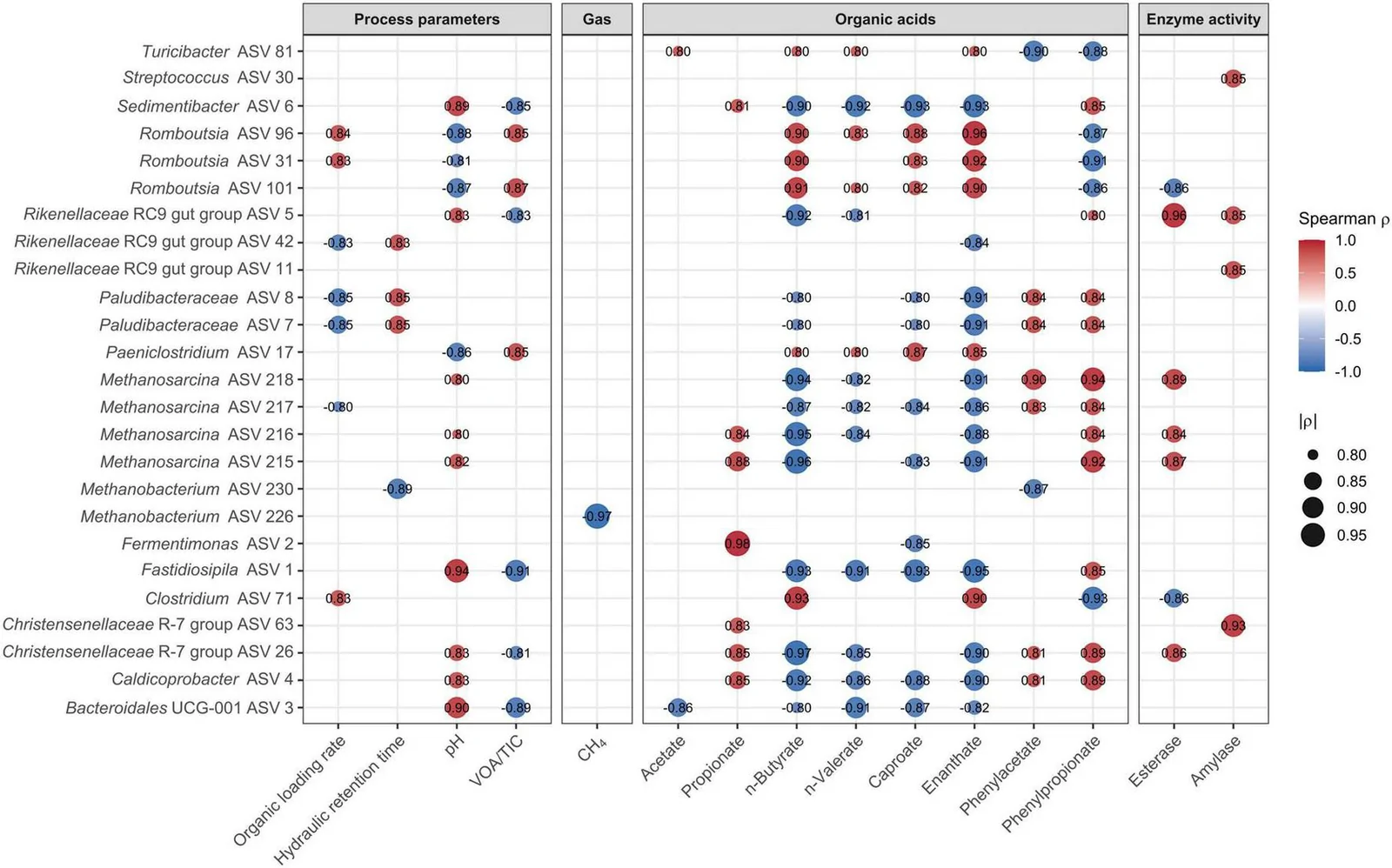
Spearman correlations between the most abundant amplicon sequence variants (ASVs) and abiotic parameters at volatile organic acid to total inorganic carbon (VOA/TIC) ratio > 1.0. Significance was defined at *p* < 0.01. Only correlations with a Spearman correlation coefficient ρ > | 0.3| are shown.

Spearman correlations between the 30 most abundant ASVs, abiotic process parameters and enzyme activities are illustrated in Figures 7, 8. Correlation analyses between dominant community members (represented by ASV relative abundance) and process parameters were split into two different datasets from timepoints reflecting VOA/TIC values below and above 1.0. This was done to limit bias for extreme values, due to large shifts in community composition at higher VOA/TIC values.

Correlations between ASV relative abundance and process parameters before the VOA/TIC ratio surpassed 1 are shown in Figure 7. Both OLR and VOA/TIC values had positive correlations with the abundance of *Oscillospiraceae* HN-HF0106 (ASV24), *Sedimentibacter* (ASV6), *Erysipeloclostridiaceae* UCG-004 (ASV13). OLR and VOA/TIC values correlated negatively with the abundance of *Tissierella* (ASV15), *Ruminiclostridium* (ASV10), *Sedimentibacter* (ASV9), *Erysipeloclostridiaceae* UCG-004 (ASV20), *Caldicoprobacter* (ASV4), *Rikenellaceae* DMER64 (ASV12), and *Rikenellaceae* (ASV16).

HRT, inversely to OLR and VOA/TIC, had positive correlations with *Tissierella* (ASV15), *Ruminiclostridium* (ASV10), *Sedimentibacter* (ASV9), *Erysipeloclostridiaceae* UCG-004 (ASV20), *Caldicoprobacter* (ASV4), *Rikenellaceae* DMER64 (ASV12), and *Rikenellaceae* (ASV16). HRT was negatively correlated to the abundance of *Oscillospiraceae* HN-HF0106 (ASV24), *Sedimentibacter* (ASV6 and ASV19), and *Erysipeloclostridiaceae* UCG-004 (ASV13).

Hydrogen sulfide (H_2_S) production shows positive correlations with the relative abundance of *Paludibacteraceae* (ASV7 and ASV8), *Sedimentibacter* (ASV6), *Methanosarcina* (ASV215, ASV216, ASV217, and ASV218), and *Fastidiosipila* (ASV1). H_2_S production correlated negatively with *Rikenellaceae* RC9 gut group (ASV5 and ASV11), *Sedimentibacter* (ASV19), *Fermentimonas* (ASV23), and *Cloacimonadales* W27 (ASV21 and ASV25).

Specific methane production had positive correlations with the relative abundance of *Tissierella* (ASV15), *Rikenellaceae* (ASV16), and *Rikenellaceae* DMER64 (ASV12). Specific methane production had a negative correlation with *Oscillospiraceae* HN-HF0106 (ASV24).

Concentrations of organic acids (propionate, *n*-butyrate, *n*-valerate, caproate, and enanthate) correlated positively with the abundances of *Oscillospiraceae* HN-HF0106 (ASV24), *Sedimentibacter* (ASV6), and *Erysipeloclostridiaceae* UCG-004 (ASV13). Negative correlations between organic acid concentrations and abundance included *Tissierella* (ASV15), *Ruminiclostridium* (ASV10), *Sedimentibacter* (ASV9), *Erysipeloclostridiaceae* UCG-004 (ASV20), *Caldicoprobacter* (ASV4), *Rikenellaceae* DMER64 (ASV12), and *Rikenellaceae* (ASV16).

Phenylacetate and phenylpropionate concentrations showed many correlations with the same ASVs. Among them were positive correlations with *Sedimentibacter* (ASV6), *Oscillospiraceae* HN-HF0106 (ASV24), *Fastidiosipila* (ASV1), and *Erysipeloclostridiaceae* UCG-004 (ASV13). They correlated negatively with the abundances of *Tissierella* (ASV15), *Sedimentibacter* (ASV9), *Ruminiclostridium* (ASV10), *Rikenellaceae* DMER64 (ASV12), *Rikenellaceae* (ASV16), and *Caldicoprobacter* (ASV4).

Amylase activity had strong and significant correlations with the relative abundance of several ASVs. The positive correlations include *Sedimentibacter* (ASV6), *Paludibacteraceae* (ASV7, ASV8), *Oscillospiraceae* HN-HF0106 (ASV24), *Fastidiosipila* (ASV1), and *Erysipeloclostridiaceae* UCG-004 (ASV13). Negative correlations were found with *Erysipeloclostridiaceae* UCG-004 (ASV20), *Fermentimonas* (ASV23), *Tissierella* (ASV15), *Rikenellaceae* DMER64 (ASV12), *Rikenellaceae* (ASV16), *Ruminiclostridium* (ASV10), and *Caldicoprobacter* (ASV4). Positive correlations with protease activity include *Tissierella* (ASV15), *Methanosarcina* (ASV216, ASV217, ASV218, and ASV219), *Fastidiosipila* (ASV1), and *Cloacimonadaceae* W5 (ASV22). Negative correlations were found with *Fermentimonas* (ASV2 and ASV23) and *Cloacimonadales* W27 (ASV14). Esterase activity showed strong and significant positive correlations with the relative abundance of *Sedimentibacter* (ASV6), *Oscillospiraceae* HN-HF0106 (ASV24), *Fermentimonas* (ASV2), and *Erysipeloclostridiaceae* UCG-004 (ASV13). Negative correlations include *Sedimentibacter* (ASV9), *Erysipelotrichaceae* (ASV20), *Tissierella* (ASV15), *Rikenellaceae* DMER64 (ASV12), *Rikenellaceae* (ASV16), *Caldicoprobacter* (ASV4), *Ruminiclostridium* (ASV10), and *Methanosarcina* (ASV217 and ASV219).

Correlations between ASV relative abundance and process parameters at VOA/TIC greater than 1.0 are shown in Figure 8. OLR correlated positively with the abundance of *Romboutsia* (ASV96 and ASV31) and *Clostridium* (ASV71). OLR correlated negatively with *Rikenellaceae* RC9 gut group (ASV42), *Paludibacteraceae* (ASV8 and ASV7), and *Methanosarcina* (ASV217). Specific methane production correlated positively with the abundance of *Methanobacterium* (ASV230).

Concentrations of some organic acids (*n*-butyrate, *n*-valerate, caproate, and enanthate) correlated positively with the abundance of *Romboutsia* (ASV 96 and ASV101) and *Paeniclostridium* (ASV17). Negative correlations include *Sedimentibacter* (ASV6), *Methanosarcina* (ASV217), *Fastidiosipila* (ASV1), *Caldicoprobacter* (ASV4), and *Bacteroidales* UCG-001 (ASV3).

Phenylacetate and phenylpropionate concentrations had positive correlations with *Paludibacteraceae* (ASV7 and ASV8), *Methanosarcina* (ASV217 and ASV218), *Christensenellaceae* R-7 group (ASV26), and *Caldicoprobacter* (ASV4). Negative correlations were found with *Turicibacter* (ASV81).

Amylase activity was positively correlated with *Streptococcus* (ASV 30), *Rikenellaceae* RC9 gut group (ASV 5 and ASV 11), and *Christensenellaceae* R-7 group (ASV63). Esterase activity was positively correlated with *Sedimentibacter* (ASV6), *Rikenellaceae* RC9 gut group (ASV5), *Methanosarcina* (ASV215, ASV216, and ASV218), and *Christensenellaceae* R-7 group (ASV26). Esterase activity correlated negatively with the abundance of *Romboutsia* (ASV101) and *Clostridium* (ASV71).

## 4 Discussion

This study explored the limits of AD process stability during increasing OLR by combined analysis of abiotic process parameters, hydrolytic enzyme activities, and microbial community dynamics, with a focus on hydrolytic functions. It is important to mention that the increasing OLR was realized by shortening the HRT, which poses a combined stress to the microorganisms in terms of organic overload and washout of slow-growing organisms. The increasing OLR led to the accumulation of VFAs, as indicated by the increasing VOA/TIC value, leading eventually to over-acidification. This process failure indicates an imbalance between the acid-producing reactions (acidogenesis) and the acid-consuming reactions (acetogenesis/methanogenesis), which could be explained by direct acid inhibition or insufficient growth rates of some methanogens and/or syntrophic VFA-oxidizing bacteria. Acid inhibition is a direct consequence of the high substrate load, while washout due to insufficient growth rates is caused by shortening the HRT. Since the latter would lead to further VFA accumulation, both effects reinforce each other, but the individual contributions of OLR increase and HRT reduction cannot be disentangled within our experimental design.

The most striking result of our experiment was that the AD process in one reactor was more robust than in the other, and withstood higher operational stress before methane production collapsed. As both reactors were operated under identical conditions, this difference in AD process resilience can only be explained by biological factors. The observed microbial divergence could be attributed to stochastic effects during community assembly such as ecological drift, since the reactors, although operated in parallel and well controlled, had a history of 847 days of parallel operation prior to the experimental period of this study. During this pre-experimental period, the AD process in both parallel CSTRs was generally stable, with VOA/TIC ratios around 0.2. Only when grass silage was loaded for the first time, VOA/TIC ratios slightly increased but remained below 0.6. Ammonium-nitrogen concentrations stayed below 4 g/L throughout the entire pre-experimental period. Although AD microbiomes are mostly shaped by deterministic factors (Peces et al., 2018), stochastic effects also play a role and have a stronger influence after process disturbance when immigrating microbes replace the indigenous populations and promote random community assembly (Yuan et al., 2019). Considering the long history of the two parallel reactors, the divergent community trajectories could also be a consequence of minor abundance differences in rare taxa (Pascual-García et al., 2025). This effect could have been mitigated by complete mixing of the reactor content before starting the experimental period. Another limitation in this context is that we had only two parallel reactors available, which limits the options to disentangle stochastic and deterministic factors that shape community assembly.

During the experimental period, an increase in OLR resulted in a slightly elevated specific methane production during the first 40 days until an OLR of 9.5 g VS/(L d). Despite the subsequent slight decrease in specific methane production until an OLR of 11.5 g VS/(L d), most physicochemical parameters showed no significant changes, indicating a stable AD process. During this time, the two microbial communities developed in parallel (Figure 5). However, the VOA/TIC ratio began to increase after the peak in specific methane production in both reactors, i.e. around day 34, and exceeded a threshold of 0.6 at an OLR 12.5 g VS/(L d). According to Scherer et al. (2021), VOA/TIC ratios below 0.3 represent a stable AD process, while values between 0.3 and 0.6 reflect a moderate level of process stability, and values above 0.6 indicate a critical state. Subsequently, ongoing increase in OLR and corresponding reduction in HRT led to process destabilization, as indicated by a pH drop below 6.0 within only a few days. Different buffer systems in the liquid phase of the AD, including bicarbonate and ammonia/ammonium, can maintain a stable pH despite ongoing accumulation of VFAs (Li et al., 2014; Sun et al., 2019; Wu et al., 2021). Once the buffer capacity was exhausted due to the accumulation of acidogenesis products at high OLR, the pH in both reactors dropped below 6.0. This sharp pH drop was defined here as the onset of severe over-acidification (in R1 after day 74 and in R2 after day 109). Notably, in both reactors, high values of the VOA/TIC ratio exceeding 1.0 coincided with severe acidification. Compared to R1, R2 exhibited a greater robustness of the AD process, taking longer for the VOA/TIC ratio to rise from 0.6 to 1.0 while maintaining methane production. The suitability of the VOA/TIC ratio as an early-warning indicator of impending acidification aligns with other studies (Moeller and Zehnsdorf, 2016; Maurus et al., 2023).

The reduction of the working volume on day 70, as a measure to cope with excessive foaming, was implemented only a few days prior to the severe over-acidification in R1 and clear divergence of the two reactor microbiomes. However, physicochemical and biological analyses indicate that differences between the reactors were already present before the volume reduction and were not induced by it. The VOA/TIC ratios and the propionate concentrations differed already earlier (Figures 1, 2), with VOA/TIC ratios of 0.50 and 0.39, and propionate concentrations of 740.9 and 519.5 mM in R1 and R2, respectively, at day 59. In addition, microbial community analysis showed differences prior to day 70, for instance, through the appearance of *Methanoculleus* in R2, which were below the 1% threshold in R1.

Specific methane production declined almost simultaneously with the severe over-acidification. In R1, it fell below 150 mL/(g VS d) after day 72, two days before the sharp pH drop, corresponding to a decrease of 30–50% compared to stable operation. This disruption in methane production was accompanied with a shift in both the bacterial and the methanogenic communities. This shift was delayed slightly in the methanogenic communities, with the bacterial shift occurring between days 74 and 78 and the methanogenic community shifting between days 85 and 91. In the bacterial community, this shift was characterized by the relative abundance of *Streptococcus*, *Paeniclostridium* and *Romboutsia* ASVs increasing above the 3% threshold, indicating that these genera can withstand the low pH values that caused the washout of other bacterial species. *Romboutsia* ASVs (ASV96 and ASV101) and *Paeniclostridium* (ASV17) were positively correlated with the concentration of C4-C7 carboxylic acids, suggesting a direct or indirect role in microbial chain elongation. Microbial chain elongation by reverse β-oxidation is an alternative electron sink in AD systems under conditions that impair methanogenesis (Wu et al., 2019). Previous studies have found *Romboutsia* and other members of the *Peptostreptococcaceae* to be associated with the production of medium-chain carboxylates in mixed-culture fermentation (Yin and Wang, 2022; Lou et al., 2025), but experimental evidence in pure-culture studies is missing.

The community shift of methanogenic archaea was characterized by the appearance of *Methanobrevibacter-*associated ASVs and the concomitant replacement of *M. thermophila* by other *Methanosarcina*-associated ASVs. The decrease in relative abundance of *M. thermophila* as a result of acidification during overloading or shock-loading was reported earlier (Lerm et al., 2012; Khafipour et al., 2020).

In R2, the sharp decline in specific methane production below 150 mL/(g VS d) occurred on day 104, six days before the severe over-acidification. This decrease in methane production was again followed by a shift in microbial community composition between days 106 and 113. As was the case in R1, the community in R2 also shifted to favor *Streptococcus*, *Paeniclostridium* and *Romboutsia* ASVs. Increasing OLR was associated with reduced HRT, which may have created a kinetic mismatch between acidogenic bacteria and slow-growing methanogenic archaea or their syntrophic partners degrading VFAs. Methanogens are considered to grow slowest in AD, having doubling times ranging from 5 to 16 days depending on environmental factors such as temperature, substrate availability, and pH (Deublein and Steinhauser, 2010). At an OLR of 11.5 g VS/(L d), the VOA/TIC ratio exceeded 0.3, indicating VFA accumulation due to the ongoing acidogenesis but impaired acetogenesis/methanogenesis. In combination with a reduced HRT of 14.6 days, this may have increased the risk of biomass washout of methanogens and/or syntrophic VFA-oxidizing bacteria. Because OLR and HRT were changed simultaneously beyond an OLR of 10.5 g VS/(L d) and an HRT of 16.6 days, their individual contributions to the observed process failure cannot be clearly distinguished.

Zhang et al. (2017) reported stable performance during mesophilic AD of chicken manure over a wide range of HRTs, while the OLR was constant at 6 g VS/(L d). After a reduction of the HRT from 20 to 10 days, VFAs began to accumulate and specific methane production to reduce, suggesting that an HRT around 10 days is a critical value. In our study, process instability indicated by a VOA/TIC ratio exceeding 0.6 occurred already at an HRT of 13.4 days, suggesting that HRT reduction alone is unlikely to explain process failure. The substantially higher OLR in the present study probably increased the susceptibility of the process to HRT reduction by imposing additional stress on the microbial community. These community perturbations may have contributed to reduced specific methane production and further VFA accumulation, ultimately resulting in over-acidification. The *mcrA* ASV data reflect only the relative composition of the methanogenic community and do not provide information about absolute cell numbers or biomass. Therefore, it cannot be distinguished whether the observed changes are driven by compositional shifts or by an overall reduction of methanogens. Likely, ASVs increasing in relative abundance may represent community members that were able to reproduce faster than others at low pH and/or at low HRT, while still decreasing in absolute abundance.

The higher robustness of the AD process in R2 against organic overloading compared to R1 was also reflected by the time course of VFA levels. VFAs are degraded by syntrophic consortia of bacteria and methanogens during the acetogenesis step. Among the VFAs, propionate is the least thermodynamically favorable for microbial degradation in methanogenic systems (Goux et al., 2015), and its degradation is often a bottleneck in AD processes (Westerholm et al., 2022). Propionate transiently accumulated in both reactors, suggesting that syntrophic propionate oxidation could not keep pace with the increasing acidogenesis rate at higher OLR, likely due to insufficient abundance or activity of syntrophic propionate-oxidizing bacteria (SPOB) or too low growth rates of SPOB and/or their methanogenic partners, leading eventually to washout at shorter HRT. Subsequent decrease in propionate concentration, accompanied by accumulation of other VFAs, suggests that propionate was metabolized via different pathways in both reactors. In R2, syntrophic propionate oxidation resumed as long as methanogenesis remained active, although the stationary propionate concentration remained higher than before the accumulation, in accordance with the higher VOA/TIC ratio that gradually reached critical values. By day 77, R1 exceeded a VOA/TIC value of 2.0, whereas the VOA/TIC value of R2 had plateaued at approximately 0.65 until day 100. This resilience might have been aided by higher bacterial diversity being retained in R2 for a longer period, illustrated by a reduction in the Shannon index in R1 from 2.8 to 1.5, while R2 retained a Shannon index of greater than 3 (Supplementary Figure 5). Higher functional redundancy, as a result of higher diversity, might have enabled the reactor community in R2 to continue syntrophic degradation of VFAs even as some community members were lost. Since ASV data cannot reveal functional information on poorly characterized taxa, metagenome sequencing would be needed to clarify which community members replaced the metabolic activities that were lost due to over-acidification and biomass washout.

In R1, the accumulation of *n*-valerate along with the decline in propionate concentration indicates that chain elongation via reverse β-oxidation may have occurred. Chain elongation requires electron donors such as ethanol or lactate (Wu et al., 2019). The transient accumulation of ethanol and lactate supports their consumption for chain elongation under methane-arrested conditions in R1. In addition to *n*-valerate formation, the sequential formation of longer-chain carboxylates (C4-C7) further supports the occurrence of reverse β-oxidation as an alternative electron sink under inhibited methanogenesis. The accumulation of *n*-butyrate along with a decline of acetate concentration may represent an initial elongation step from acetate, but *n*-butyrate is also a direct acidogenesis product that accumulates when syntrophic butyrate oxidation is not possible due to methanogenesis inhibition. However, the accumulation of caproate indicates chain elongation of *n*-butyrate, while enanthate indicates further elongation of *n*-valerate. The stepwise increase in C4, C5, C6, and C7 carboxylates is consistent with the prevalence of reverse β-oxidation, in which short-chain carboxylates are elongated by acetyl-CoA provided by the oxidation of acidogenesis products such as ethanol or lactate. Several ASVs presented positive correlations with the concentrations of medium-chain carboxylates, suggesting their direct or indirect role in chain elongation of VFAs. One such ASV, *Oscillospiraceae* HN-HF0106 (ASV24), belongs to a family that also harbors genera known as chain elongating bacteria, such as *Caproicibacter*, *Caproicibacterium*, and *Caproiciproducens* (Flaiz et al., 2020; Gu et al., 2021). Genome-centric metagenome analyses are needed to confirm the metabolic functions of the bacteria represented by this ASV. *Clostridium* sp. (ASV71) correlated positively with *n*-butyrate and enanthate concentrations. This ASV is affiliated with the genus *Clostridium sensu stricto* 1, which also comprises species involved in chain elongation (Ji et al., 2022).

High concentrations of lactate and second peaks of ethanol and acetate concentrations towards the end of the experimental phase in R1 indicate that chain elongation was eventually also inhibited. As lactic acid bacteria (LAB) are highly acid-tolerant (Anumudu et al., 2024) and have high growth rates, it can be expected that they maintained fermentation activity until the end. One ASV identified as a potential LAB is *Streptococcus* (ASV30). This ASV increased in relative abundance in both reactors after acidification. The genus *Streptococcus* comprises facultatively aerobic and homofermentative cocci that produce lactic acid as a major end product of glucose fermentation (du Toit et al., 2014).

In R2, severe over-acidification occurred at a later stage, and potential products of chain elongation were observed only towards the end of the experiment. This indicates a delayed metabolic shift compared to R1, consistent with the prolonged maintenance of methanogenic activity. In R2, *M. thermophila* retained high relative abundance until severe acidification, even increasing in relative abundance up to acidification. Hence, we can speculate that the biological cause of the deviating process performance in both reactors is likely associated with differences in the composition of the bacterial communities and particularly the syntrophic VFA-degrading bacteria. Slight differences in low abundant taxa during earlier experimental periods or in the pre-experimental period could have resulted in different trajectories of the bacterial community dynamics. Further increasing the OLR while simultaneously shortening the HRT imposed stronger selection pressure on microbial communities as reflected by the decreasing trend in alpha-diversity (Supplementary Figure 5), leading to the dominance of fast growing and copiotrophic competitors. At an OLR of 12.5 g VS/(L d), the communities in R1 and R2 diverged from each other. Divergence of microbial communities during elevated OLR or stronger selection pressure is a common phenomenon that was also observed by Goux et al. (2015). In the present study, this resulted in collapse of the AD process in R1, whereas the community in R2 retained the methane production capacity longer. Accordingly, the bacterial community in R2 kept a higher diversity compared to R1.

Propionate-induced over-acidification as the result of elevated OLR has been reported previously. For instance, Lerm et al. (2012) observed this effect in a near-thermophilic (47 °C) AD process using sewage sludge and rapeseed oil as substrates. In their study, the OLR was up to 12-fold increased via shock loads, resulting in high VFA concentrations and a decrease in pH. Initially, propionate concentrations increased, but were later exceeded by acetate concentration. Similar observations were reported by Khafipour et al. (2020), where shock loading of propionate in a stable mesophilic AD process using fresh dairy manure led to initial high propionate accumulation followed by increasing acetate concentrations. In contrast, Goux et al. (2015) and Li et al. (2015) reported different VFA patterns in mesophilic AD under increasing OLR. In their studies, acetate was the dominant VFA during stepwise OLR increases from 1.6 to 4.5 g VS/(L d) using sugar beet pulp as a substrate, and from 3 to 12 g VS/(L d) using rice straw and pig manure, respectively. In both cases, propionate accumulated more slowly and persisted longer. A similar trend was described by Ahring et al. (1995) in a thermophilic CSTR experiment, where increasing OLR from 4.2 g VS/(L d) to 6.3 g VS/(L d) using pig and cow manure as substrates resulted to an overall increase in VFA concentrations with acetate dominance and more persistent propionate. Similarly, in a full-scale AD using cattle manure and energy crops, acetate was the dominant VFA during over-acidification, with 13–21-fold higher concentrations than propionate (Moeller and Zehnsdorf, 2016). These findings demonstrate that over-acidification during organic overload in AD does not always follow the same pattern and can be dominated by different VFAs. The predominant VFA during over-acidification can serve as an indicator of the underlying metabolic limitation. While propionate accumulation indicates impaired hydrogenotrophic methanogenesis or insufficient activity of SPOB, acetate accumulation suggests inhibition of acetoclastic methanogenesis or syntrophic acetate oxidation, with the former being dominant in mesophilic AD (Zhao et al., 2018).

Although physicochemical parameters showed no significant changes until day 40, hydrolytic enzyme activities increased in response to the rising OLR, with a first maximum coinciding with the peak in specific methane production between OLR 7.5 and 9.5 g VS/(L d). Higher OLR enables faster growth and increased metabolic activity, which requires higher enzyme activities. Zuo et al. (2013) reported similar observations in a two-stage AD system using vegetable waste, where the highest hydrolytic enzyme activities occurred at the highest OLR of 2.6 g VS/(L d). Similarly, Kardos et al. (2009) observed in a study of a sewage treatment plant that hydrolytic enzyme activities react more quickly to altering OLR than VFA concentrations, as VFA concentrations reflect the balance between production and consumption rather than immediate substrate degradation. However, with increasing OLR, corresponding decrease in HRT, and rising VOA/TIC ratio, hydrolytic enzyme activities began to diverge. Protease activity declined first, whereas amylase activity showed a temporary decrease and rose to a second maximum. Esterase activity fluctuated between 0.2 and 0.7 U/mL, showing a slight increase with increasing OLR up to 12.5 g VS/(L d), whereas its final drop to non-detectable activities occurred after severe acidification had already taken place.

Differences in hydrolytic enzyme activities between the two reactors occurred mainly after the over-acidification had started in R1. Although increased hydrolytic activity is expected to enhance the availability of fermentable substrates, the delayed onset of over-acidification in R2 indicates efficient downstream processes of fermentation products. Accordingly, higher hydrolytic activity was not associated with reduced stability but rather reflected an enhanced capacity of the system to cope with increasing OLR and shorter HRT. Notably, R2, which maintained high or increasing hydrolytic enzyme activities, did experience over-acidification at a later stage. This ability to maintain higher hydrolytic enzyme activity might have been supported by enrichment of fast reproducing hydrolytic microbes. One difference between the two reactors was a steady increase in relative abundance of ASVs associated with the *Rikenellaceae* RC9 gut group in R2. This taxon represents non-cultured fermentative bacteria, which have been found in mammalian gut microbiomes and discussed to be associated with VFA regulation in swine gut communities (Wu et al., 2024). At VOA/TIC > 1, *Rikenellaceae* RC9 gut group (ASV 5) abundance had strong positive correlations with esterase and amylase activities. It is possible that this ASV represents fast reproducing hydrolytic microbes.

To our knowledge, only a few studies investigated the relationship between process stability and hydrolytic enzyme activities, primarily in two-stage AD. Gasch et al. (2013) found significant positive correlations between esterase activity and methane content as well as COD in a two-stage AD system and suggested esterase activity as a monitoring parameter for AD plants. Zuo et al. (2013) reported a decline in amylase activity in the acidogenic reactors of a two-stage AD system using vegetable waste under organic overload at an OLR of 2.6 g VS/(L d). The results of the present study indicate that the observations in two-stage AD are only partially transferable to single-stage AD using energy crops. In our study, esterase activity slightly increased with rising OLR and declined sharply after severe over-acidification, but did not provide additional information of process dynamics or the onset of process disturbances under the conditions applied in this study. However, the decline in amylase activity during over-acidification under organic overload also occurred earlier in R1 than in R2. Furthermore, the prolonged maintenance of elevated hydrolytic function in R2 suggests a higher resilience and adaptation to operational stress. The observed relationship between sustained hydrolytic activity and adaptation is supported by findings from Jensen et al. (2021). Although their study investigated hydrolytic enzyme activities in single-stage AD using lignocellulosic feedstock, they likewise found that changes in cellulase and xylanase activities coincided with shifts in specific microbial groups during substrate adaptation. These findings indicate that hydrolytic enzyme dynamics are closely linked to microbial adaptation under changing operational conditions.

Correlation analysis supports the observation that protease activity was the earliest to decrease under organic overload or washout of proteolytic organisms due to shorter HRT, as it exhibited the only negative correlation coefficient (Supplementary Figure 4). Hecht and Griehl (2009) reported that during mesophilic AD of kitchen waste with high protein and fat contents and a gradually increasing OLR, phenylacetate and phenylpropionate accumulated. These compounds are formed during the AD of the aromatic amino acids phenylalanine, tyrosine, and tryptophan. Similarly, Müller et al. (2016) observed increased concentrations of these compounds when high dosages of protease were added to a mesophilic AD of cattle manure, chicken dung, and maize silage. Furthermore, the addition of proteases led to a decrease in biogas production, suggesting that the aromates formed through enhanced protein degradation cause process inhibition. In our study, higher levels of phenylacetate and phenylpropionate were also detected (Supplementary Figure 3), which supports the findings of previous studies. The accumulation of these intermediates began, when protease showed increased activity and coincided with its subsequent decline. Reduced protease activity accompanied by an increased VOA/TIC value indicates inhibition of proteolytic microorganisms during VFA accumulation. This suggests that proteolytic microorganisms exhibit a high sensitivity to increasing VFAs. On the other hand, they could have been washed out due to decreasing HRT. Reduced protease activity may also be associated with an overall reduction in active microorganisms.

Enzyme activity was correlated to the abundance of individual ASVs present in digestate samples at given times. In several cases, ASVs belonging to the same genus had opposite correlations with the same enzyme activity measurements. For example, ASVs affiliated to *Sedimentibacter* (ASV6 and ASV9) were either positively or negatively associated with esterase activity. One could speculate that these individual ASVs represent species or strains with different functional potential in the AD community, but metagenome-assembled genomes or other omics data would be necessary to validate this assumption.

Hydrolytic enzyme measurements were restricted to free extracellular enzymes. Intracellular hydrolytic enzymes, as well as hydrolases bound to extracellular polymeric substances (EPS) or the cell wall, were not assessed. Previous studies reported higher activities of free extracellular hydrolases compared to cell-bound enzymes (Parawira et al., 2005). In contrast, Zumstein and Helbling (2019) observed that a substantial proportion of enzyme activity is associated with EPS-bound hydrolases or intracellular fractions, which were not captured in this study. Notably, Parawira et al. (2005) measured polysaccharide-degrading enzymatic activities (e.g., amylase, xylanase), whereas Zumstein and Helbling (2019) examined proteolytic activities (peptidase, protease). This suggests that the fraction captured by measuring free extracellular enzymes also may depend on the type of enzyme. Nevertheless, activity measurements should be interpreted relatively rather than as absolute values. A more comprehensive picture of hydrolytic enzymes could be retrieved by proteome analyses, which would also cover enzyme complexes attached to the cells, such as cellulosomes, and intracellular hydrolases.

Overall, stepwise increase in OLR and corresponding decrease in HRT initially resulted in a slight increase in specific methane production, coinciding with elevated hydrolytic enzyme activities. This was followed by distinct responses in the two reactors, induced by changing operational conditions. R1 underwent over-acidification, accompanied by a shift toward chain elongation as the prevailing community function, while R2 maintained methanogenesis as the dominant community function under more severe operational conditions. Correlation analysis between hydrolytic enzyme activities and the VOA/TIC ratio (used as an indicator for process stability) showed that only protease activity exhibited a negative relationship. Changes in microbial community composition reflected increasing operational stress. Higher bacterial diversity appeared to enhance process stability, preventing AD process failure for a longer time in one reactor than in the other. Correlations revealed distinct associations between specific ASV abundances, process parameters, enzyme activities, and metabolites. *Romboutsia* (ASV96 and ASV101) and *Paeniclostridium* (ASV17) were associated with C4-C7 carboxylates, whereas *Clostridium* sp. (ASV71) was positively correlated with *n*-butyrate and enanthate. An ASV affiliated with *Rikenellaceae* RC9 gut group (ASV5) was positively correlated to esterase and amylase activities, and coincided with process stability under stressful operational parameters. Our results show that maintaining a diverse microbial community is important for resilient AD processes. In conclusion, the diverging reactor responses despite identical operational changes highlight that even small differences in community composition or stochastic effects may influence functional adaptation and process resilience. To improve resolution of microbial community structure and potential functions, future work should include a more detailed functional analysis of the microbiome using genome-centric metagenomics and omics methods that show the actual metabolic activity under specific process conditions, such as metatranscriptomics or metaproteomics.

## Data Availability

The microbial community sequencing datasets presented in this study can be found in online repositories. The names of the repository/repositories and accession number(s) can be found at: https://www.ebi.ac.uk/ena, PRJEB113461.
