# Supplementary material for: Effect of increasing organic loading rate on hydrolytic enzyme activities and microbial communities during anaerobic digestion of energy crops
Source: Front Microbiol. 2026 Aug 12;17:1894607. doi: 10.3389/fmicb.2026.1894607 (PMC13584728; doi:10.3389/fmicb.2026.1894607)
Supplement: Supplementary file 1 [file Data_Sheet_1.pdf]

Effect of increasing organic loading rate on hydrolytic enzyme activities and microbial communities during anaerobic digestion of energy crops

Frederik Bade[1*], Elliot Guerra-Blackmer[2], Alberto Meola[3, 4], Simon Hellmann[3, 5], Sören Weinrich[3, 6], Lucie Moeller[1]*, Sabine Kleinsteuber[2]

[1]Helmholtz Centre for Environmental Research – UFZ, Department Systemic Environmental Biotechnology, Leipzig, Germany

[2]Helmholtz Centre for Environmental Research – UFZ, Department of Microbial Biotechnology, Leipzig, Germany

[3]DBFZ Deutsches Biomasseforschungszentrum gemeinnützige GmbH, Leipzig, Germany

[4]Leipzig University, Faculty of Mathematics and Computer Science, Leipzig, Germany

[5]Chemnitz University of Technology, Chair of Automatic Control and System Dynamics, Chemnitz, Germany

[6]Münster University of Applied Sciences, Faculty of Energy, Building Services and Environmental Engineering, Steinfurt, Germany

*** Correspondence:**

Frederik Bade
frederik.bade@ufz.de

Lucie Moeller
lucie.moeller@ufz.de

Supplementary Material

1 Supplementary Data

The amplicon sequence variant (ASV) dataset is provided as a separate Excel File (Supplementary Table S1).

2 Supplementary Figures

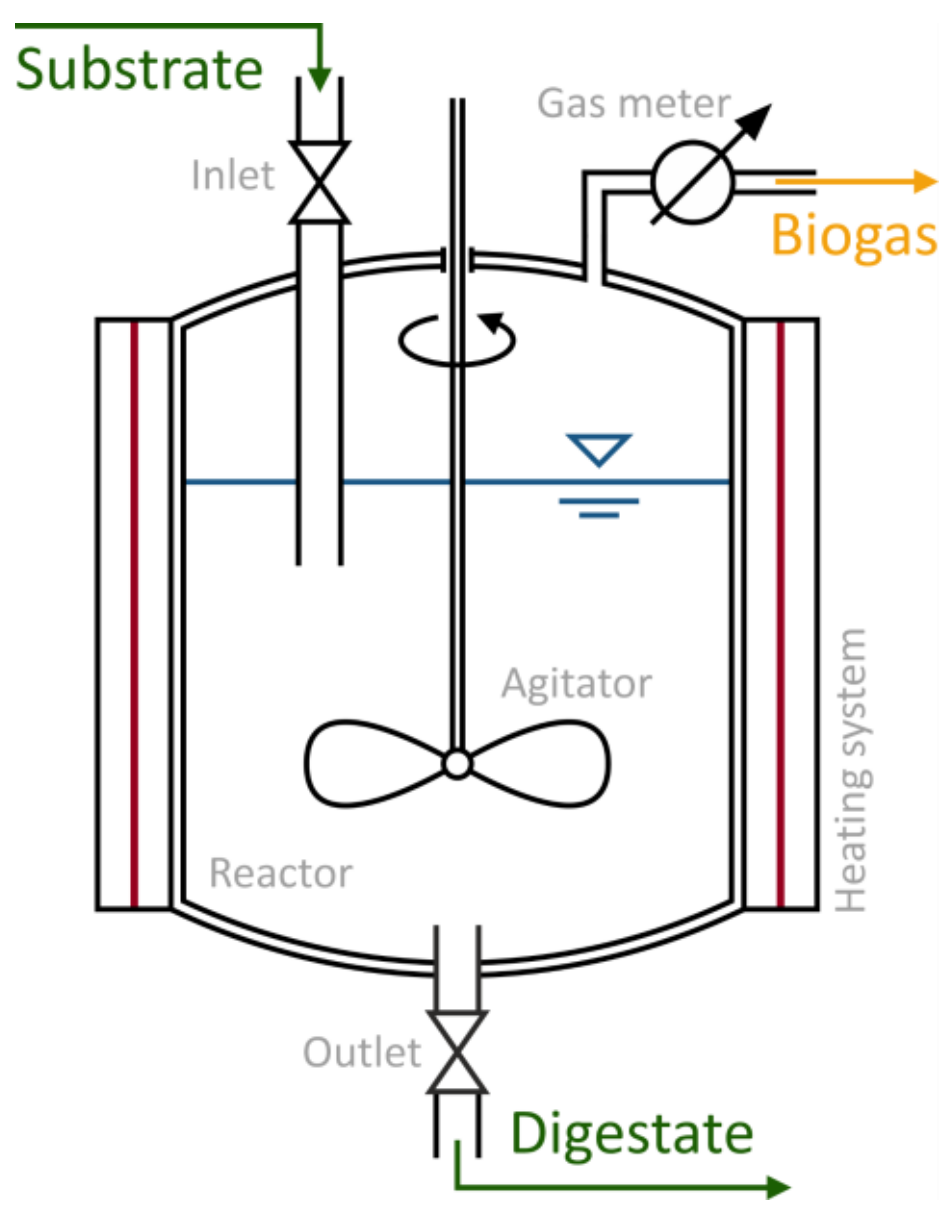

Figure S1. Schematic diagram of one of the continuous stirred tank reactors (CSTRs) used in the present study. Two identical CSTRs, with 12-L initial working volume, were operated under identical conditions.

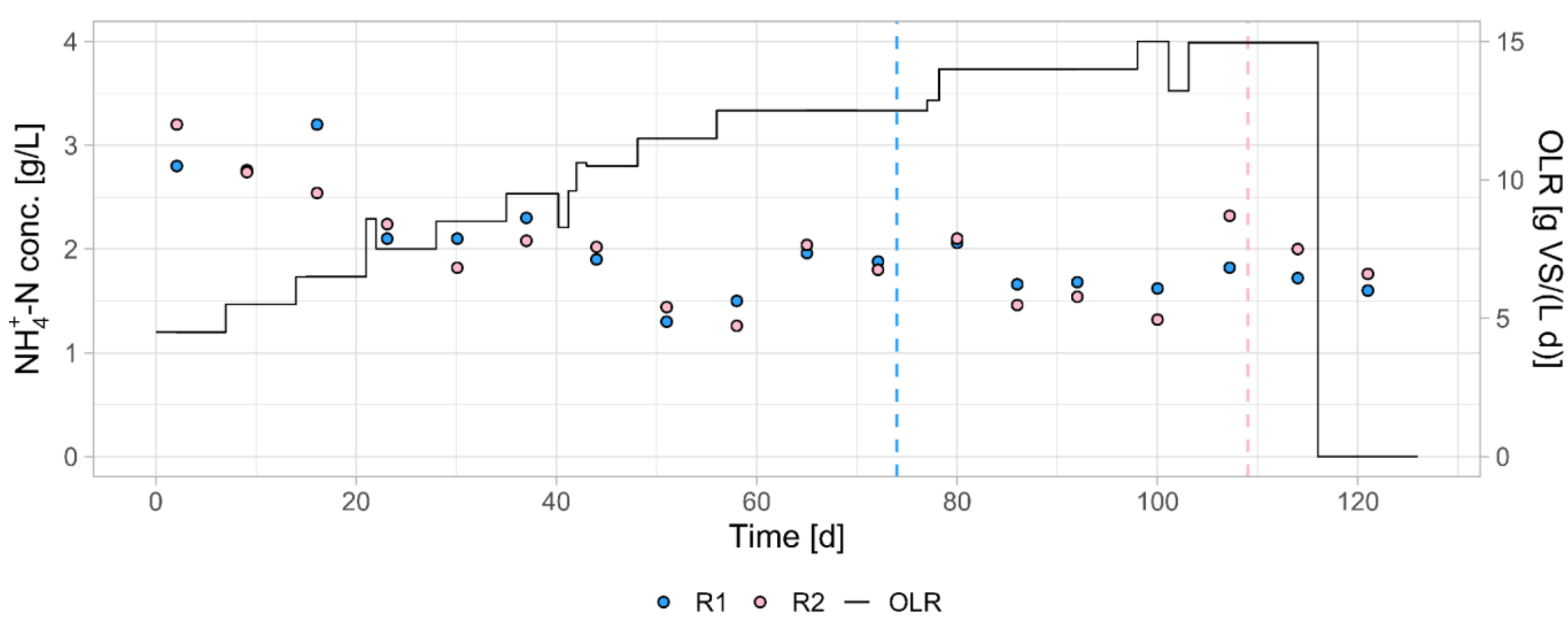

Figure S2. Time courses of ammonium nitrogen concentration in the two parallel CSTRs (R1 and R2) along the gradual increase of the organic loading rate (OLR). Dashed vertical lines represent the onset of over-acidification, defined as the last sampling point with pH>6.0 (blue line: R1, pink line: R2).

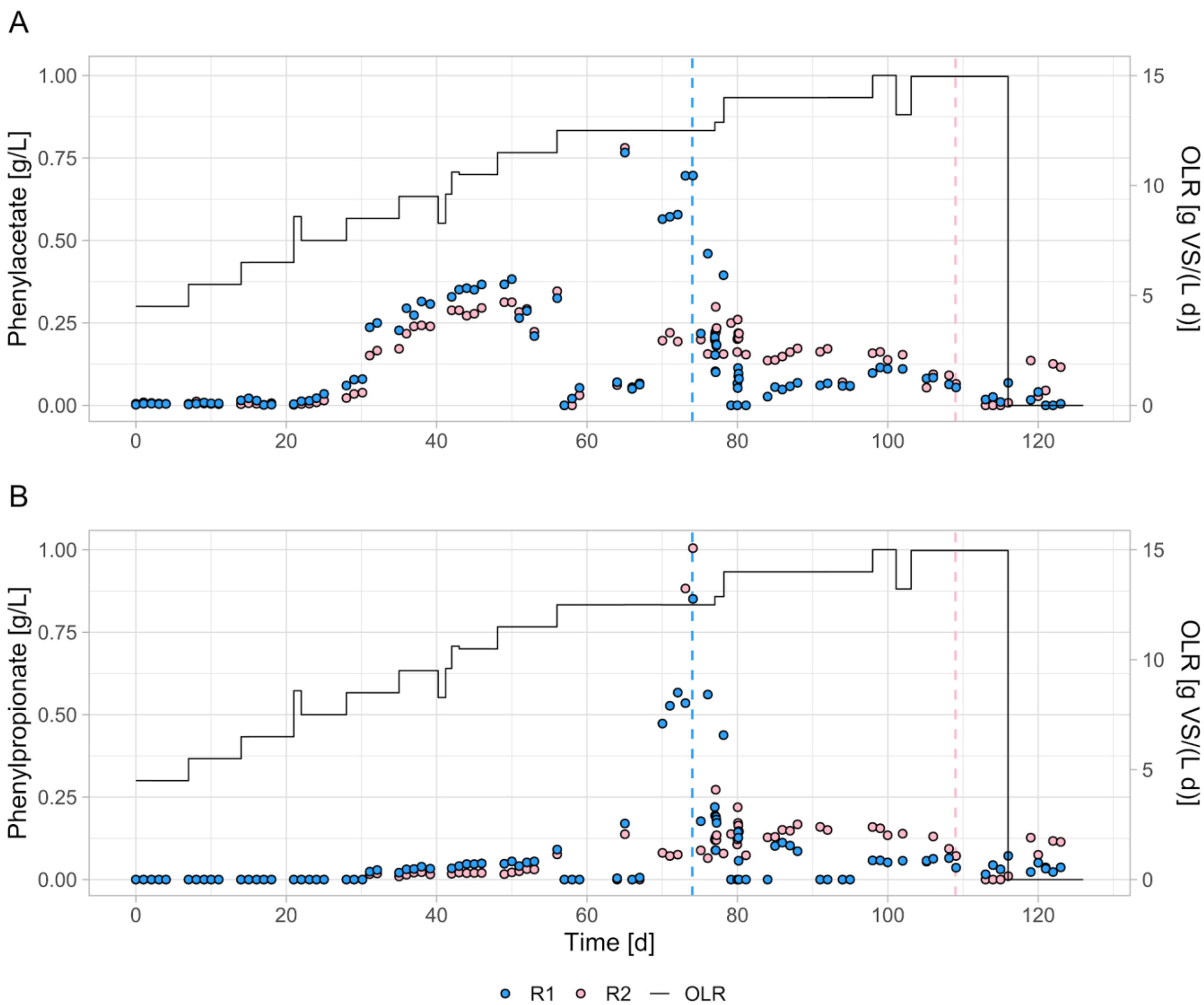

Figure S3. Time courses of phenylacetate (A) and phenylpropionate (B) concentrations in the two parallel CSTRs (R1 and R2) along the gradual increase of the organic loading rate (OLR). Dashed vertical lines represent the onset of over-acidification, defined as the last sampling point with pH>6.0 (blue line: R1, pink line: R2).

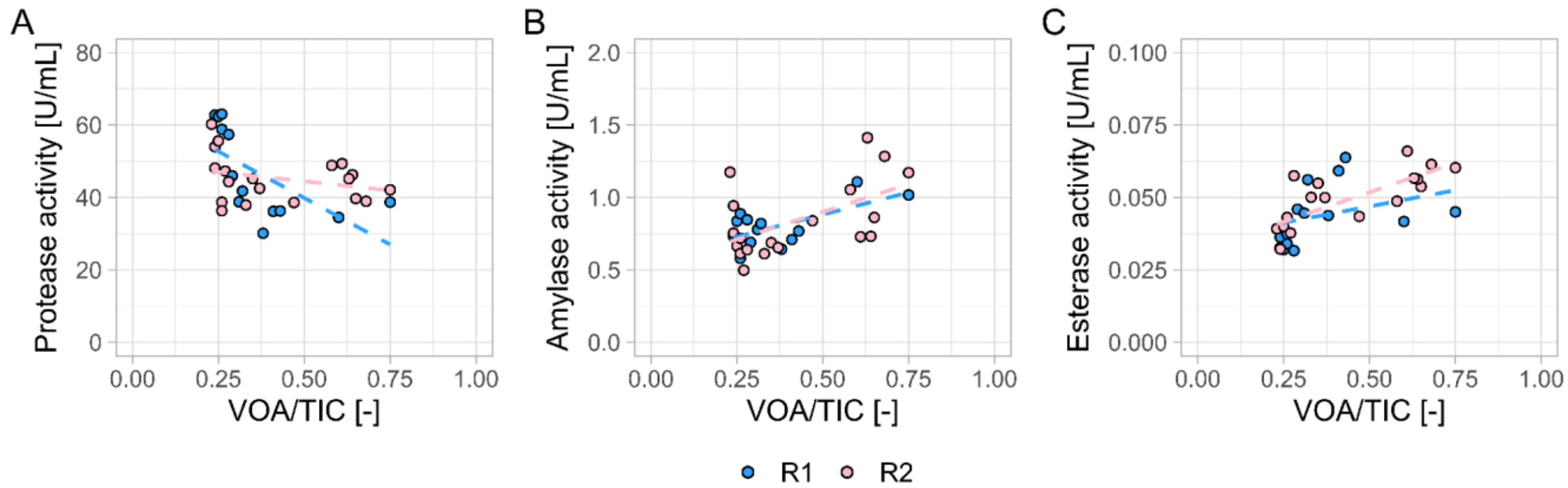

Figure S4. Spearman correlation between enzyme activities and VOA/TIC values in reactors R1 and R2. Regression lines are included for visualization purposes only to illustrate the monotonic relationships.

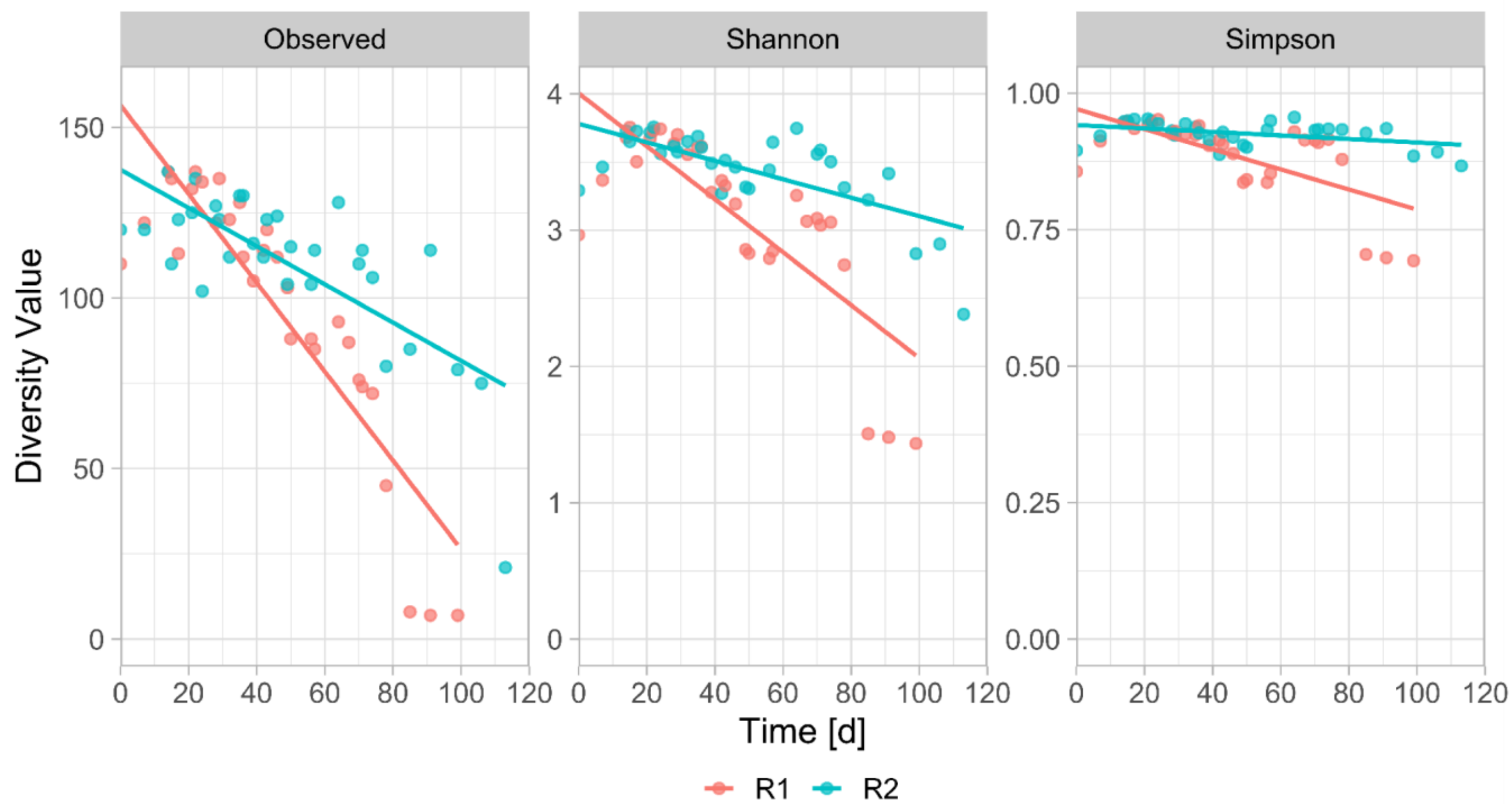

Figure S5. Alpha diversity metrics for 16S rRNA amplicon sequence variant (ASV) data, indicating the trends of richness (Observed ASV number), Shannon index, and Simpson index of bacterial communities.

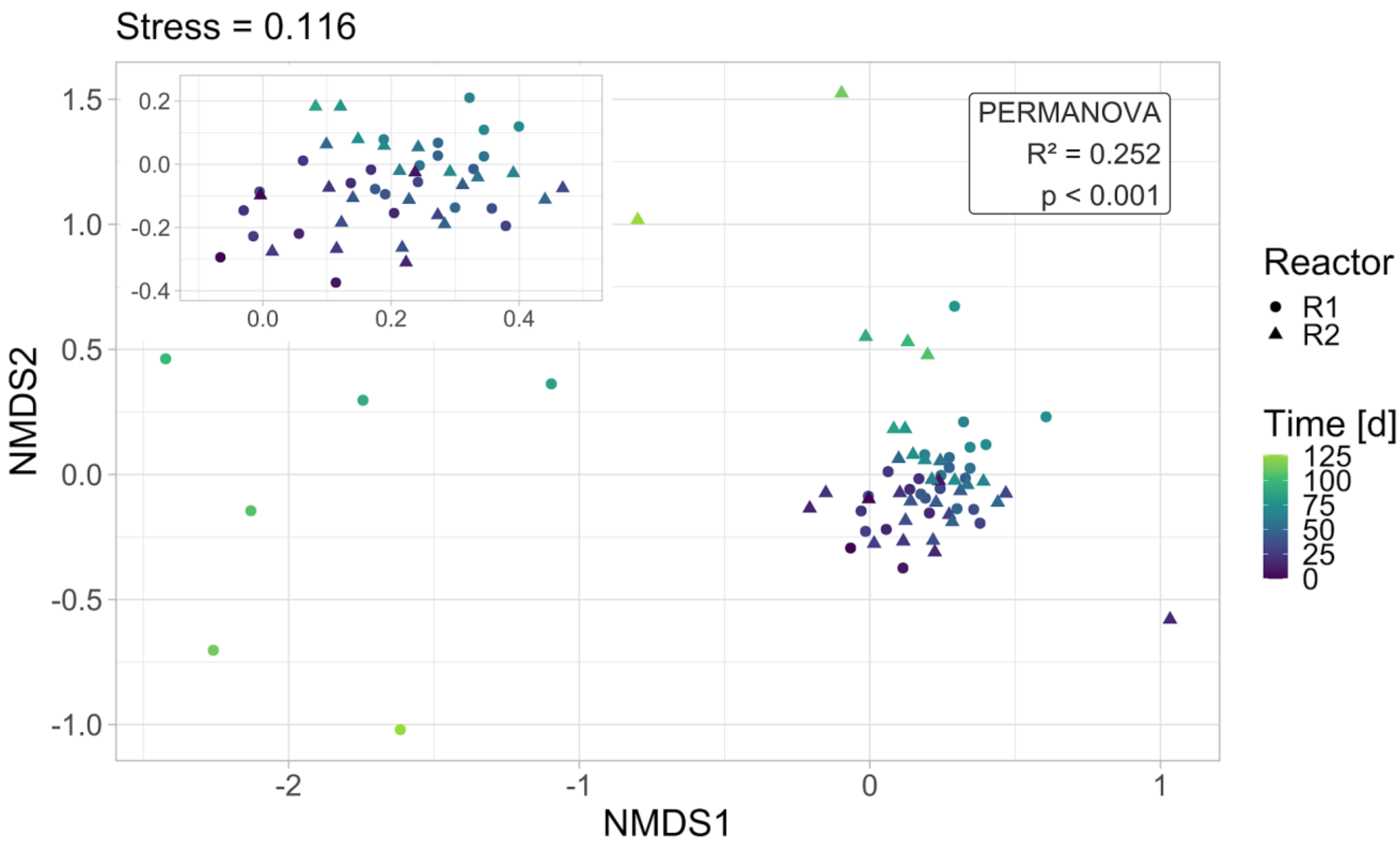

Figure S6. Non-metric multidimensional scaling (NMDS) plot of Bray-Curtis dissimilarity of methanogenic communities based on *mcrA* ASV data.
